# Cryo-EM visualization of RAD51 filament assembly and end-capping by XRCC3-RAD51C-RAD51D-XRCC2

**DOI:** 10.1126/science.aea1546

**Published:** 2026-02-26

**Authors:** Luke A. Greenhough, Lorenzo Galanti, Chih-Chao Liang, Simon J. Boulton, Stephen C. West

**Affiliations:** 1 DNA Recombination and Repair Laboratory The Francis Crick Institute, London, NW1 1AT, UK; 2 DSB Metabolism Laboratory The Francis Crick Institute, London, NW1 1AT, UK

## Abstract

The five RAD51 paralogs play critical roles in the recombinational repair of DNA breaks and replication fork maintenance. Mutations in the genes that encode them cause heritable breast and ovarian cancers, and the cancer-prone disease Fanconi anemia. Here, we show that the RAD51 paralogs form two distinct heterotetrameric complexes, RAD51B-RAD51C-RAD51D-XRCC2 (RAD51B complex) and XRCC3-RAD51C-RAD51D-XRCC2 (XRCC3 complex). The RAD51B complex assembles RAD51 nucleoprotein filaments by a dynamic ATP hydrolysis–dependent mechanism. In contrast, substitution of RAD51B with XRCC3 confers distinct properties to the XRCC3 complex, which assembles and stably caps the 5′-termini of RAD51 filaments on single-stranded DNA, tailed duplex DNA and D-loop intermediates, as visualized by cryoelectron microscopy. Highly conserved across evolution, the XRCC3 complex reveals new insights into RAD51 filament formation and capping during DNA repair and replication fork stabilization.

Homologous recombination (HR) is a critical cellular pathway that repairs DNA double-strand breaks and safeguards genome stability by protecting and restarting broken replication forks (1). Central to this process is the assembly of RAD51 nucleoprotein filaments onto Replication Protein A (RPA)–coated single stranded DNA (ssDNA). RAD51 polymerizes onto ssDNA with a pitch of approximately 100 Å and 6 protomers per helical turn, binding DNA primarily via the conserved L1 and L2 DNA-binding loops. These filaments mediate homologous pairing with the sister chromatid to enable homology-directed repair, replication fork reversal, and fork restart (2–6).

The assembly of RAD51 filaments is tightly regulated by tumor suppressors, most notably the BRCA2-PALB2 complex and the RAD51 paralogs (RAD51B, RAD51C, RAD51D, XRCC2 and XRCC3) (7). BRCA2 functions as a molecular chaperone, delivering RAD51 to ssDNA via RAD51-binding motifs — BRC repeats and a C-terminal RAD51 filament binding (CRB) domain — and OB folds that bind ssDNA (8–17). PALB2 promotes the chromatin localization of BRCA2-RAD51 complexes (18, 19), whereas the FIGNL1-FIRRM complex negatively regulates RAD51 by actively disassembling RAD51 filaments through ATP-dependent translocation (20).

The RAD51 paralogs share between 20-30% sequence similarity with RAD51. Heritable mutations in the RAD51 paralog genes, as also found with *BRCA2*, are associated with hereditary breast and ovarian cancers (21–24). Mutations in *RAD51C* and *XRCC2* are also implicated in the cancer-prone syndrome Fanconi anemia (25, 26). Loss of the RAD51 paralogs results in embryonic lethality (27–31), and their cellular depletion causes severe phenotypes, including growth defects, impaired sister chromatid recombination, reduced RAD51 focus formation at DNA damage sites, and hypersensitivity to DNA crosslinking agents and PARP inhibitors (32). RAD51 and its paralogs are also essential for replication fork protection and restart (33–36). Together, these findings underscore the indispensable roles the RAD51 paralogs play in maintaining RAD51 filament stability and function during DNA repair and replication stress.

Biochemical and structural analyses revealed that RAD51B, RAD51C, RAD51D, and XRCC2 assemble into a tetrameric complex known as BCDX2, which selectively binds ssDNA and actively promotes RAD51 filament formation (37). This process is driven by the coordinated ATPase activities of RAD51B and RAD51C and facilitated by conformational flexibility of the C-terminal RecA-like domain of RAD51B (38–40). In addition, RAD51C also interacts directly with XRCC3 to form the RAD51C-XRCC3 (CX3) complex (37, 41), but its precise biochemical functions in DNA repair and replication fork maintenance remain elusive. Here, we show that CX3 interacts with RAD51D and XRCC2 to form a heterotetrameric complex comprising XRCC3-RAD51C-RAD51D-XRCC2. By combining cryoelectron microscopy (cryo-EM) with single molecule and biochemical assays, we define the structure and function of this previously uncharacterized complex in the context of DNA double-strand break repair and replication fork stabilization and restart.

## Heterotetrameric XRCC3-RAD51C-RAD51D-XRCC2 assembles RAD51 filaments

**Fig. 1:**
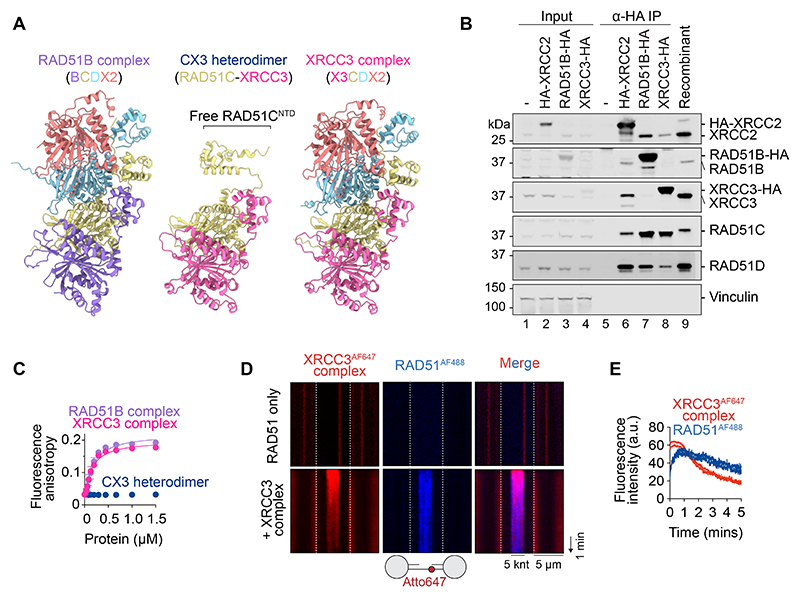
XRCC3 complex assembles RAD51 on ssDNA. **(A)** AlphaFold3 models of the RAD51B complex (RAD51B-RAD51C-RAD51D-XRCC2), RAD51C-XRCC3 (CX3 heterodimer) and putative XRCC3 complex (XRCC3-RAD51C-RAD51D-XRCC2). **(B)** Immunoprecipitation (α-HA) of RAD51 paralog complexes from HeLa Kyoto cell lines stably overexpressing HA-XRCC2, RAD51B-HA or XRCC3-HA. **(C)** ssDNA binding by RAD51B complex, XRCC3 complex or CX3 in the presence of ADP.AlFx, measured by changes in fluorescence anisotropy. **(D)** Representative kymographs showing the selective binding of RAD51^AF488^ (blue) to ssDNA in the absence and presence of the XRCC3^AF647^ complex (red). The gapped DNA substrate is indicated below. White dashed lines indicate bead-DNA boundaries. **(E)** Fluorescence intensities of XRCC3^AF647^ complex and RAD51^AF488^ signal over time. n=6. n values represent the number of independent ssDNA molecules. Shaded area = SEM. a.u. = arbitrary units.

AlphaFold3 modeling (42) of both BCDX2 and CX3 revealed that RAD51B and XRCC3 engage a common interface on RAD51C (Fig. 1A). However, within CX3, and in contrast to BCDX2, the N-terminal domain (NTD) of RAD51C remains unengaged. These insights led us to explore whether CX3 might associate with RAD51D and XRCC2 to form a tetrameric XRCC3-RAD51C-RAD51D-XRCC2 complex analogous to RAD51B-RAD51C-RAD51D-XRCC2, as predicted by AlphaFold (Fig. 1A). For simplicity, we will refer to these two putative RAD51 paralog heterotetramers as the XRCC3 and RAD51B complexes, according to their unique subunit.

To determine the subunit constitution of RAD51 paralog complexes in human cells, we immunoprecipitated complexes from HeLa Kyoto cells stably overexpressing either hemagglutinin (HA)–tagged RAD51B, XRCC3 or XRCC2 (as a control). As expected, RAD51B-HA pulled down RAD51C, RAD51D and XRCC2, indicative of the heterotetrameric RAD51B complex (Fig. 1B, lane 7). Consistent with the AlphaFold prediction, XRCC3-HA pulled down RAD51C, RAD51D and XRCC2, rather than just RAD51C (lane 8), demonstrating that human cells indeed contain two heterotetrameric RAD51 paralog assemblies: the RAD51B and XRCC3 complexes. Confirming its presence in both XRCC3 and RAD51B complexes, HA-XRCC2 (lane 6) co-immunoprecipitated all five RAD51 paralogs.

The identification of the XRCC3 complex aligns with functional genetic studies, in which knockout of *XRCC3, RAD51C, RAD51D*, or XRCC2 results in a strong phenotype, in contrast to the milder, non-lethal phenotype observed in *RAD51B* knockouts (fig. S1A) (32). Additionally, it is consistent with the absence of RAD51B in certain amphibians (e.g., *Xenopus tropicalis*) and several teleost fish, unlike all components of the XRCC3 complex which are universally conserved across vertebrates (fig. S1B). Likewise, all components of the XRCC3 complex, but not RAD51B, are present in *Drosophila melanogaster* (43).

To permit structure–function analyses of the XRCC3 complex, we co-expressed XRCC3, RAD51C, RAD51D and XRCC2 in baculovirus-infected insect cells and purified it to homogeneity (fig. S2A). The XRCC3 complex bound ssDNA with an affinity comparable to the RAD51B complex, as determined by fluorescence anisotropy, whereas the CX3 heterodimer failed to bind ssDNA at the same protein concentrations (Fig. 1C).

To determine the function of the XRCC3 complex, RAD51^AF488^, RPA^AF555^ and the XRCC3^AF647^ complex were fluorescently labeled with AlexaFluor (AF) fluorophores (fig. S2B), and single-molecule analyses were performed using the Lumicks C-trap. Specific binding to ssDNA was visualized using a gapped DNA substrate in which 5 knt (kilo nucleotides) of ssDNA was flanked by 6.4 kbp (kilo base pairs) and 6.3 kbp double-stranded DNA (dsDNA) segments (fig. S2C). In the absence of RPA, RAD51^AF488^ was selectively recruited by the XRCC3^AF647^ complex to the ssDNA gapped region (Fig. 1D). As RAD51 is known to bind ssDNA and dsDNA with comparable affinities (2), these results show that the XRCC3 complex promotes the specific binding of RAD51 to ssDNA. At the zero time point, RAD51 and XRCC3 complex bind simultaneously to the ssDNA, followed by a gradual decrease in binding (Fig. 1E). These results were confirmed by direct visualization of RAD51 filament formation on ssDNA by negative stain electron microscopy (fig. S2D-F). The XRCC3 complex increased both filament number and length, indicating a role in RAD51 filament nucleation and extension.

To investigate RAD51 filament assembly in the presence of RPA, single molecule analyses were used to monitor the displacement of RPA^AF555^ upon the binding of RAD51^AF488^ to λ-ssDNA. We found that, in contrast to RAD51B complex (38), the XRCC3 complex was unable to stimulate RAD51 assembly on RPA-coated ssDNA, as indicated by total AF488 signal intensity and force measurements following RAD51 polymerization (fig. S2G-I). In this respect, the RAD51B and XRCC3 complexes show a fundamental difference in their ability to assemble RAD51 on ssDNA, consistent with *in vivo* data indicating a non-redundant role in RAD51 filament assembly (32).

### The XRCC3 complex caps RAD51 filaments

**Fig. 2:**
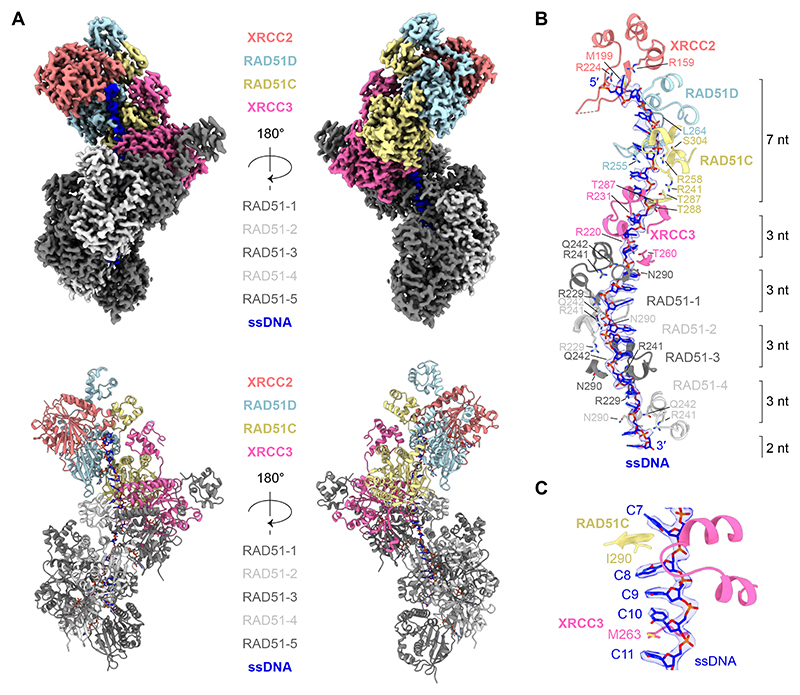
Cryo-EM visualization of XRCC3 complex capping RAD51-ssDNA filaments. **(A)** Cryo-EM structure of the XRCC3 complex capping the end of a RAD51-ssDNA filament. Top, cryo-EM map (2.7 Å). Bottom, atomic model. **(B)** Cryo-EM density of ssDNA (blue) and key interacting residues in RAD51, XRCC3, RAD51C, RAD51D and XRCC2. Full 2D interaction map shown in fig. S5A. **(C)** Cryo-EM density of ssDNA (blue) bound to XRCC3, stacked into a triplet through hydrophobic residues RAD51C^I290^ and XRCC3^M263^.

We next assembled RAD51 filaments onto ssDNA in the presence of the XRCC3 complex, ATP and divalent cations (Mg^2+^ and Ca^2+^), and analyzed the resulting complexes by single-particle cryo-EM. Although compositional heterogeneity was observed, with filament lengths ranging from two to six RAD51 protomers (fig. S3), subsequent classification and refinement yielded a 2.7 Å reconstruction of a presynaptic filament composed of five RAD51 protomers associated with the XRCC3 complex (Fig. 2A, fig. S4A). Remarkably, the XRCC3 complex caps the 5’-end of the RAD51 filament, with XRCC3 directly interacting with RAD51, and XRCC2 forming the cap.

Continuous cryo-EM density corresponding to the full length of the ssDNA (21 nt) was observed, running 5′ to 3′ from XRCC2 toward XRCC3 - mirroring the polarity previously observed in the RAD51B complex (Fig 2B, fig. S5A) (38, 39). RAD51 engages the ssDNA through a triplet-binding mode, with RAD51 residues N290 and Q242 contacting the first phosphate of each nucleotide triplet. Arginine residues in the L1 loop, R241 and R229, interact with the second and third phosphates, respectively, as observed with equivalent residues in XRCC3 (R231, R220) and RAD51C (R258, R249). Triplet stacking — a hallmark of filament activation that aids homology search — is facilitated by RAD51^V273^ of the L2 loop, which inserts between nucleotide triplets. Equivalent hydrophobic residues in XRCC3 (M263) and RAD51C (I290) perform the same role, stabilizing a nucleotide triplet bound to XRCC3 (Fig. 2C, fig. S5B).

In contrast, ssDNA bound by RAD51C, RAD51D, and XRCC2 — comprising seven nucleotides of ssDNA — does not exhibit triplet stacking and instead adopts a conformation identical to that observed in the RAD51B complex (39). Here, the ssDNA interacts with basic patches formed by side chains in the L1 and L2 loops (fig. S5C). Cytosine bases are stabilised by hydrogen bonds and cation-π interactions with RAD51C (T288, S256), RAD51D (Q220, Q253), and XRCC2 (R159), while the phosphodiester backbone is engaged via hydrogen bonds and salt bridges mediated by RAD51C (T287, T288, R249, R258, S304), RAD51D (L264), and XRCC2 (Q200, R224). Several of these DNA-interacting residues correspond to variants of uncertain significance (VUS) catalogued in the ClinVar database (fig. S5A). Notably, RAD51C^R258^, which contacts the DNA backbone, is mutated to histidine in Fanconi anemia (26), while XRCC2^R159^, which engages a cytosine base, is reported as R159C/H in the ClinVar database. In agreement with equivalent mutations in the RAD51B complex (38, 39), RAD51C^R258H^ or XRCC2^R159A^ mutations reduce the ssDNA binding affinity of the XRCC3 complex (fig. S5D).

### Evolutionary conservation of RAD51^NTD^ remodeling

RAD51 and its paralogs share a common domain architecture, containing an NTD and RecA-like C-terminal domain (CTD), connected by a linker and alpha-helix 5 (α5). XRCC2 is the exception in that it lacks an NTD (fig. S6A). RAD51 protomers interact via a conserved interface, where the linker and α5 of one protomer engage the adjacent protomer, positioning phenylalanine 86 (RAD51^F86^) into a hydrophobic pocket (fig. S6B). The NTD of RAD51 (RAD51^NTD^) adopts an *in cis* configuration such that it interacts with the CTD of the same protomer (fig. S6C).

**Fig. 3:**
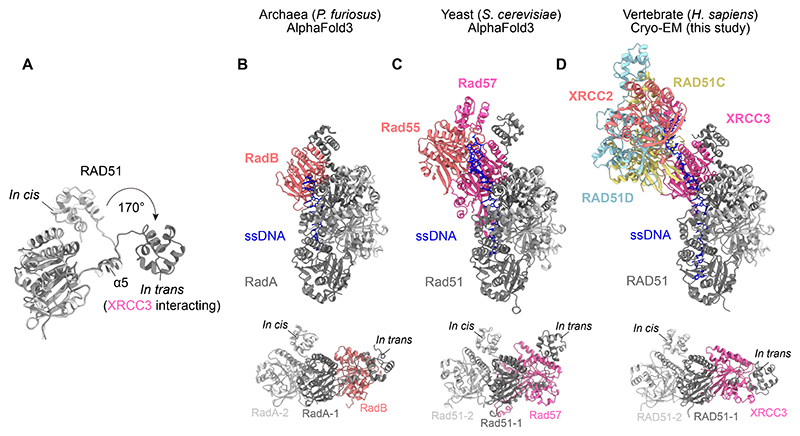
Conservation of RAD51^NTD^ remodeling. **(A)** Aligned atomic models of RAD51-1 and RAD51-2 showing the 170° rotation of RAD51^NTD^ from *in cis* to an *in trans* configuration by XRCC3 interaction. AlphaFold3-predicted atomic models: **(B)** *Pyrococcus furiosus* RadB capped RadA-ssDNA filaments. **(C)** *Saccharomyces cerevisiae* Rad55-Rad57 capped Rad51-ssDNA filaments. **(D)** Cryo-EM solved atomic model of human XRCC3-RAD51C-RAD51D-XRCC2 capped RAD51-ssDNA filament. Bottom panels show top-down views of RadA, Rad51 and RAD51 N-terminal domain rearrangements upon interaction with RadB, Rad57 and XRCC3.

RAD51-XRCC3 interactions are not driven hydrophobically, as an equivalent pocket is missing in the CTD of XRCC3 (XRCC3^CTD^). Furthermore, we find that the RAD51^NTD^ now binds *in trans* to engage XRCC3^CTD^, undergoing a 170° rotation from its usual *in cis* configuration (Fig. 3A, fig. S6B-C). The interaction is electrostatically driven, as glutamate residues in RAD51 (E50 and E59) form salt bridges with arginine residues in XRCC3 (R204 and R174, respectively) (fig. S6D). Both the XRCC3 complex and CX3 bind RAD51 with comparable affinities (fig. S6E-F), in contrast with the RAD51B complex which engages only transiently with RAD51 on ssDNA (38). To probe the basis of these differences, we compared the XRCC3^CTD^-RAD51 interface with a model of the RAD51B^CTD^-RAD51 interface. We observed that the RAD51^NTD^ engages with an electropositive region on XRCC3^CTD^, whereas the corresponding region in RAD51B^CTD^ lacks these positively charged residues, precluding a stable interaction between RAD51B^CTD^ and RAD51^NTD^ (fig. S6G).

To investigate whether the mode of RAD51 interaction observed in the XRCC3 complex is evolutionarily conserved, we used AlphaFold3 (42) to predict structures of the archaeal (RadB) and yeast (Rad55-Rad57) paralogs capping RadA and Rad51 filaments, respectively (Fig. 3B-C, fig. S6H-I), and compared these with our cryo-EM structure of the XRCC3 complex capping the RAD51-ssDNA filament (Fig. 3D). Despite considerable divergence, we identified several key conserved structural features. Firstly, RadB, Rad55, and XRCC2 all lack the NTDs found in their respective recombinases (RadA, Rad51 and RAD51) enforcing filament capping by preventing further protomer addition. Secondly, RadB, Rad57, and XRCC3 each engage their respective recombinases in a manner that rearranges each proximal protomer NTD into an *in trans* configuration. These findings support a conserved mechanism of RAD51 paralog function and are consistent with the proposed orthology between Rad55/XRCC2 and Rad57/XRCC3 (43). RAD51B orthologs have not been identified in archaea and yeast, indicating the importance of the XRCC3 complex throughout evolution.

### RAD51C ATPase is uncoupled in the XRCC3 complex

**Fig. 4:**
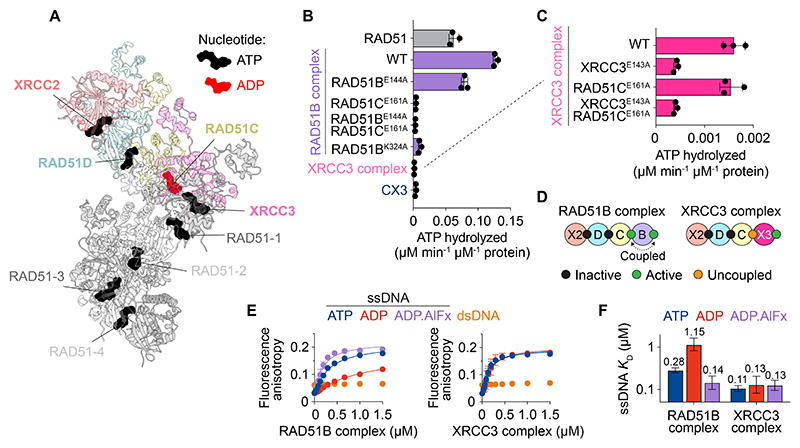
Nucleotide binding and hydrolysis activities of the XRCC3 complex. **(A)** Atomic model of XRCC3 capped RAD51-ssDNA filament highlighting bound ATP and ADP. **(B)** ATP hydrolysis by RAD51, RAD51B complex (wildtype, RAD51B^E144A^, RAD51C^E161A^, RAD51B^E144A^RAD51C^E161A^), XRCC3 complex and CX3. **(C)** ATP hydrolysis by the XRCC3 complex and its catalytic mutants (XRCC3^E143A^, RAD51C^E161A^, XRCC3^E143A^RAD51C^E161A^). **(D)** Schematic summarizing ATPase activities of the RAD51B and XRCC3 complexes. Inactive denotes ATP binding but not hydrolysis. Active denotes ATP binding and hydrolysis. Uncoupled denotes ADP-trapped. **(E)** ssDNA binding by the RAD51B and XRCC3 complexes, in the presence of ATP, ADP and ADP.AlFx, or dsDNA. **(F)** Quantification of ssDNA binding affinities of the RAD51B and XRCC3 complexes in the presence of ATP, ADP and ADP.AlFx.

Seven molecules of ATP and one molecule of ADP were unambiguously built into the cryo-EM map (Fig. 4A, fig. S7A). RAD51D and XRCC2 were both bound to ATP, as observed in the RAD51B complex, but neither is catalytically active due to the absence of an otherwise conserved catalytic glutamate residue (fig. S7B). XRCC3 and RAD51 protomers were also bound to ATP, and the presence of Ca^2+^ in their active sites reveal these ATPases to be inhibited. Unexpectedly, RAD51C bound to ADP, despite vitrification in the presence of ATP and Ca^2+^.

To investigate this further, the ATPase activities of the XRCC3 complex were compared with those of the RAD51B complex (Fig. 4B-C). XRCC3 complex displayed minimal ATPase activity relative to the RAD51B complex, and residual activity was eliminated by an XRCC3 catalytic glutamate mutant (XRCC3^E143A^), confirming that XRCC3′s active site is catalytically competent. Surprisingly, mutation of the catalytic glutamate in RAD51C (RAD51C^E161A^) had no effect on ATPase activity, despite the same mutation abolishing the ATPase activity in the RAD51B complex. Prior work showed that ATP hydrolysis by RAD51C depends on coupling to RAD51B, specifically through a lysine finger (K324) in the mobile RAD51B^CTD^ (38). However, an equivalent lysine finger is absent in the XRCC3^CTD^ (fig. S7C), eliminating ATPase transactivation. This difference results in the uncoupling of RAD51C ATPase activity, explaining the stable trapping of ADP by RAD51C (Fig. 4B-D).

The coupled RAD51B-RAD51C ATPase in the RAD51B complex modulates its affinity for ssDNA, such that binding is greatest in the presence of ATP (or the transition state mimetic ADP.AlFx), and lowest in the presence of ADP (Fig. 4E-F). Due to the uncoupled nature of the RAD51C ATPase in the XRCC3 complex, however, the binding affinities remain unchanged in the presence of different nucleotides. Thus, the RAD51B complex transiently associates with ssDNA through ATP hydrolysis, in contrast to the XRCC3 complex which stably engages ssDNA independent of ATP hydrolysis.

### XRCC3-directed RAD51 assembly on tailed duplex DNA

**Fig. 5:**
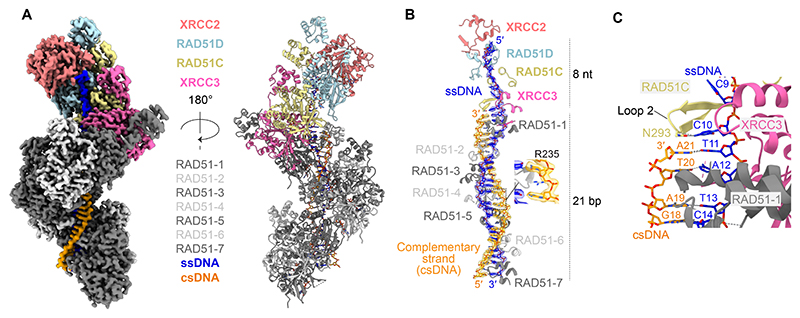
Cryo-EM visualization of XRCC3 complex capping RAD51 on 5′ tailed duplex DNA. **(A)** Cryo-EM structure of the XRCC3 complex capping the end of a RAD51 filament assembled on duplex DNA containing a 5′ ssDNA overhang. Left, cryo-EM map (2.6 Å). Right, corresponding atomic model rotated 180°. **(B)** Cryo-EM density and atomic model highlighting the ssDNA (blue) and complementary strand DNA (csDNA, orange). The 5′ ssDNA overhang is engaged by RAD51C, RAD51D, and XRCC2, whereas the 21-bp duplex DNA is bound by XRCC3 and RAD51. Inset figure showing RAD51^R235^ triplet nucleotide intercalation. **(C)** Atomic model of the XRCC3-bound triplet, showing a hydrogen bond between RAD51C^N293^ and ssDNA base cytosine 10 (C10).

Having established a role for the XRCC3 complex in capping RAD51-ssDNA filaments, we next used cryo-EM to investigate the structure of the XRCC3 complex and RAD51 bound to duplex DNA containing a 5′ ssDNA overhang. We resolved a 2.6 Å cryo-EM map of the XRCC3 complex and seven RAD51 protomers together bound to 8 nt ssDNA and 21 bp duplex DNA (Fig. 5A, fig. S4B, fig. S8). RAD51C, RAD51D, and XRCC2 bound to the 5′ ssDNA overhang via interactions described for the association of XRCC3 complex with ssDNA (Fig. 2A-B). By contrast, XRCC3 and the RAD51 filament bound to the duplex DNA, stabilized by π-cation-π stacking interactions from RAD51^R235^ nucleotide triplet intercalation (Fig. 5B) (5). As such, XRCC3 functionally mimics RAD51 by binding a nucleotide triplet, arranged via hydrophobic residues RAD51C^I290^ and XRCC3^M263^, and facilitating base pairing with homologous DNA. Intriguingly, the first nucleotide of the triplet does not pair with complementary DNA but instead hydrogen bonds with RAD51C^N293^ within the RAD51C L2 loop (Fig. 5C). The carboxamide group of asparagine can both donate and accept hydrogen bonds and is therefore able to interact with all four bases. This interaction appears to serve dual functions: stabilizing the duplex-ssDNA junction and gating duplex extension by blocking the propagation of base-pairing towards RAD51C-RAD51D-XRCC2.

### D-loop stimulation and structure

**Fig. 6:**
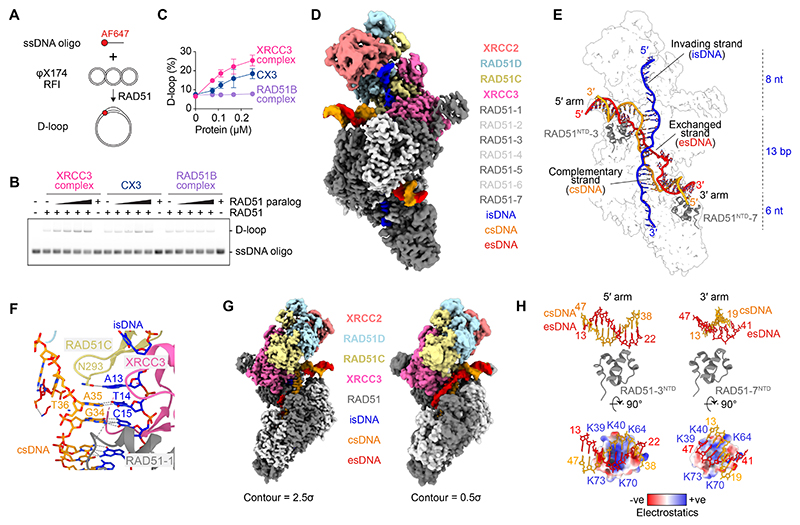
D-loop formation by XRCC3 complex and RAD51. **(A)** Schematic of the RAD51 D-loop assay. AF647 = AlexaFluor647. **(B)** Representative agarose gel from a D-loop assay, comparing stimulation activities of the XRCC3 complex, the CX3 heterodimer and RAD51B complex. **(C)** Quantification of D-loop assay shown in (B). **(D)** Cryo-EM structure (3 Å resolution) of the XRCC3 complex capping a RAD51 D-loop. **(E)** Outline of cryo-EM map and atomic model of DNA for the XRCC3 complex-capped D-loop presented in (D). The invading strand DNA (isDNA), complementary strand DNA (csDNA) and exchanged strand DNA (esDNA) are highlighted. **(F)** Atomic model of 5′ junction, showing base pairing between RAD51C^N293^ and isDNA adenine 13. **(G)** Cryo-EM density of the XRCC3 complex-capped RAD51 D-loop at contour levels 2.5σ and 0.5σ, revealing the lower resolution displaced strand (esDNA). **(H)** Atomic model of the N-terminal domains (NTDs) of RAD51-3 and RAD51-7 and their associated 5′ and 3′ duplex DNA arms, respectively. Electrostatic surface potential representation showing an electropositive patch formed by RAD51 residues K39, K40, K64, K70 and K73.

Given the structural similarity between XRCC3 and RAD51, we hypothesized that XRCC3 may enhance RAD51’s recombinase activity by stabilizing key recombination intermediates. To test this, we measured the ability of XRCC3 to stimulate RAD51-mediated displacement (D)-loops between ssDNA and homologous plasmid DNA (Fig. 6A). The XRCC3 complex, and to a lesser extent the CX3 complex, stimulated RAD51-mediated strand invasion, whereas the RAD51B complex showed no activity (Fig. 6B-C), further supporting the functional divergence between RAD51B and XRCC3 complexes.

To gain further insights into this process, we used cryo-EM to visualize filaments on a D-loop. RAD51 filaments were assembled on ssDNA (invading strand: isDNA) in the presence of the XRCC3 complex and then incubated with partially homologous duplex DNA. The duplex DNA comprised a complementary strand (csDNA) homologous to the isDNA and a heterologous strand (esDNA) mimicking the displaced strand of the D-loop. Homology was restricted to a central 15-bp region, flanked by homologous 5′ and 3′ duplex arms (defined as the 5′ and 3′ arms relative to the isDNA) (fig. S9A) (44–46).

The resulting 3 Å cryo-EM structure revealed a stable D-loop containing the XRCC3 complex, seven RAD51 protomers and clear cryo-EM density attributable to the isDNA, csDNA and esDNA (Fig. 6D–E, fig. S4C, fig. S10). Of the 15 nucleotides of homology provided by the invading ssDNA, 13 were fully base paired with complementary DNA and resolved matching a +2 registry (fig. S9B). In the paranemic joint, two base pairs were bound by XRCC3, three triplet base pairs by RAD51-1 to -3, and two base pairs by RAD51-4 (fig. S9C). RAD51C, RAD51D and XRCC2 contacted the upstream 5′ isDNA, while RAD51-5 and -6 contacted the downstream 3′ isDNA (Fig. 6E). Within each triplet, RAD51^R235^ stabilizes base stacking through π-cation-π intercalation as well as the unpaired nucleotide within the RAD51-4 triplet. Likewise, and as observed in the tailed duplex structure, RAD51C^N293^ binds the unpaired nucleotide from the XRCC3-bound triplet (Fig. 6F) – a feature not observed in RAD51-only D-loops (fig. S11A-C) (45). These results provide a key explanation for D-loop stimulation by the XRCC3 complex and CX3.

RAD51 achieves strand exchange through three distinct DNA binding sites. The primary site binds the isDNA which pairs complementarily with the csDNA during synapsis. A second site, composed of residues K284, R303, K304, R306, and K313, captures the esDNA, which mimics the displaced strand of the D-loop (fig. S11D). In the XRCC3 complex-capped RAD51 D-loop, only four nucleotides of the esDNA bound this site (fig. S9D) while the remainder exhibited weak cryo-EM density due to the absence of equivalent residues in RAD51C or XRCC3 (Fig. 6G). The third DNA binding site resides in the RAD51^NTD^, comprising electropositive patches formed by lysine residues (K39, K40, K64, K70, K73) (fig. S11E). These patches on the RAD51-3^NTD^ and RAD51-7^NTD^ associated with the 5′ and 3′ arms of duplex DNA respectively (Fig. 6H). At the 3′ junction, where the esDNA becomes displaced and the csDNA pairs with the isDNA, RAD51^F279^ (from the L2 loop) stacked its aromatic ring against the terminal base pair of the 3′ arm, as observed in the RAD51-only D-loop (fig. S9E, fig. S11F).

## Discussion

This work addresses a longstanding gap in our understanding of the five RAD51 paralogs and challenges the prevailing paradigm that they assemble into the BCDX2 tetramer and CX3 dimer (37). Instead, we find that the paralogs form two distinct tetrameric assemblies, each containing RAD51C, RAD51D, and XRCC2, with either RAD51B or XRCC3 conferring overlapping but functionally divergent roles. We propose that RAD51 paralog function is fundamentally organized into a RAD51 filament assembly-promoting RAD51B complex and a capping XRCC3 complex, a division of labor that spans homologous recombination and replication-associated repair.

All five RAD51 paralogs are important in establishing RAD51 foci formation in human cells following DNA damage (32). By selectively assembling RAD51 on ssDNA, they restrict inappropriate filament assembly on dsDNA – a known source of genomic instability (20, 47, 48). The RAD51B complex functions as a recombination mediator, promoting RAD51 filament assembly on RPA-coated ssDNA (38, 39). This occurs through an ATP hydrolysis-dependent mechanism in which the protein alternates between high and low affinity ssDNA binding modes that facilitate filament nucleation and extension. In contrast, the XRCC3 complex targets RAD51 to RPA-free ssDNA, lacks ATPase coupling and consequently forms a stable cap at the 5′ termini of RAD51 filaments. The absence of an NTD in XRCC2 restricts the successive loading of additional RAD51 protomers at the 5′-end of the filament, enforcing filament growth and disassembly at the 3′ end, consistent with previous proposals (36). Given that double-strand break repair is initiated from a 3′-tailed DNA following resection, this 5′-3′ polarity of filament assembly ensures that the filament is stably established and extended towards the 3′-terminus.

Structural modelling of archaeal RadB and yeast Rad55-Rad57 revealed striking similarities in filament capping, indicating a universally conserved mechanism from archaea to metazoans. The absence of RAD51B orthologs in several vertebrates (such as amphibians and fish), *Drosophila melanogaster*, yeast, and archaea suggests that XRCC3-containing orthologous complexes perform a conserved ancestral function that predates the emergence of RAD51B. This pattern supports the idea that RAD51B evolved later to perform separate and additional roles in RAD51 filament assembly.

Capping by the XRCC3 complex not only stabilizes RAD51 filaments but also enhances their strand exchange activity. We observed that the XRCC3 complex, but not the RAD51B complex, stimulates D-loop formation by RAD51, as also reported for the orthologous *S. cerevisiae* Rad55-Rad57 complex (49). Our cryo-EM structure of the XRCC3 complex assembled with RAD51 on a D-loop revealed base pairing between complementary DNA sequences to form a paranemic joint — a key intermediate in the search of homology. Within this structure, we found that interactions between XRCC3 and RAD51 led to remodeling of the terminal RAD51^NTD^ into an *in trans* configuration. Similar remodeling was also observed in the cryo-EM reconstructions containing ssDNA and tailed duplex DNA substrates, and in AlphaFold3 predictions of archaeal and yeast RAD51 paralog capping complexes. The relocation of the first RAD51^NTD^ may facilitate duplex DNA engagement during homology search or provide a platform for accessory factors that support strand pairing.

RAD51 and its paralogs also play key roles in the response to replication stress (6, 33, 35, 50). Uncoupling of the replicative helicase and polymerase upon encountering DNA lesions results in accumulation of RPA-bound ssDNA behind the fork (51). Fork remodeling by RAD51, helicases and translocases into a four-way ‘chicken foot’ structure, exposes a 5′ ssDNA overhang that may be capped and stabilized by XRCC3 complex to prevent nucleolytic degradation by MRE11 (52, 53). In addition, RAD51-mediated strand exchange contributes to both fork reversal (via strand invasion into the nascent lagging strand) and fork restart (via invasion into the parental duplex) in processes that are likely to be facilitated by the XRCC3 complex (6, 33, 34). Cells lacking *RAD51C, RAD51D, XRCC2* and *XRCC3* show high sensitivity to the crosslinking agent mitomycin C and to the PARP inhibitor olaparib, whereas *RAD51B*-deficient cells exhibit less acute sensitivity (32). This distinction suggests that the XRCC3 complex plays a particularly prominent role in responding to replication-associated damage, where fork stability and protection are paramount.

In conclusion, these findings revise long-standing models of RAD51 paralog function and highlight a central role for the XRCC3 complex in safeguarding genome stability during both recombination and replication stress.

## Supplementary Material

MDAR Reproducibility Checklist

Supplementary Material

## Data Availability

Cryo-EM density maps and atomic models of XRCC3 complex in association with RAD51 on ssDNA, tailed duplex DNA and D-loops will be deposited at the Electron Microscopy Data Bank (EMDB) prior to manuscript acceptance. PDB accession codes will be provided. All other data and materials reported here are available on request.
