# Supplementary material for: Cryo-EM visualization of RAD51 filament assembly and end-capping by XRCC3-RAD51C-RAD51D-XRCC2
Source: Science. Author manuscript; Available in PMC 2025 Nov 26. (PMC7618403; doi:10.1126/science.aea1546)
Supplement: Supplementary Material [file EMS210956-supplement-Supplementary_Material.pdf]

Science AAAS

Supplementary Materials for

Cryo–electron microscopy visualization of RAD51 filament assembly and end-capping by XRCC3-RAD51C-RAD51D-XRCC2

Luke A. Greenhough *et al.*

Corresponding author(s): Luke A. Greenhough, luke.greenhough@crick.ac.uk; Stephen C. West, stephen.west@crick.ac.uk

DOI: 10.1126/science.aea1546

The PDF file includes:

Figs. S1 to S13
Table S1
References

Other Supplementary Material for this manuscript includes the following:

MDAR Reproducibility Checklist

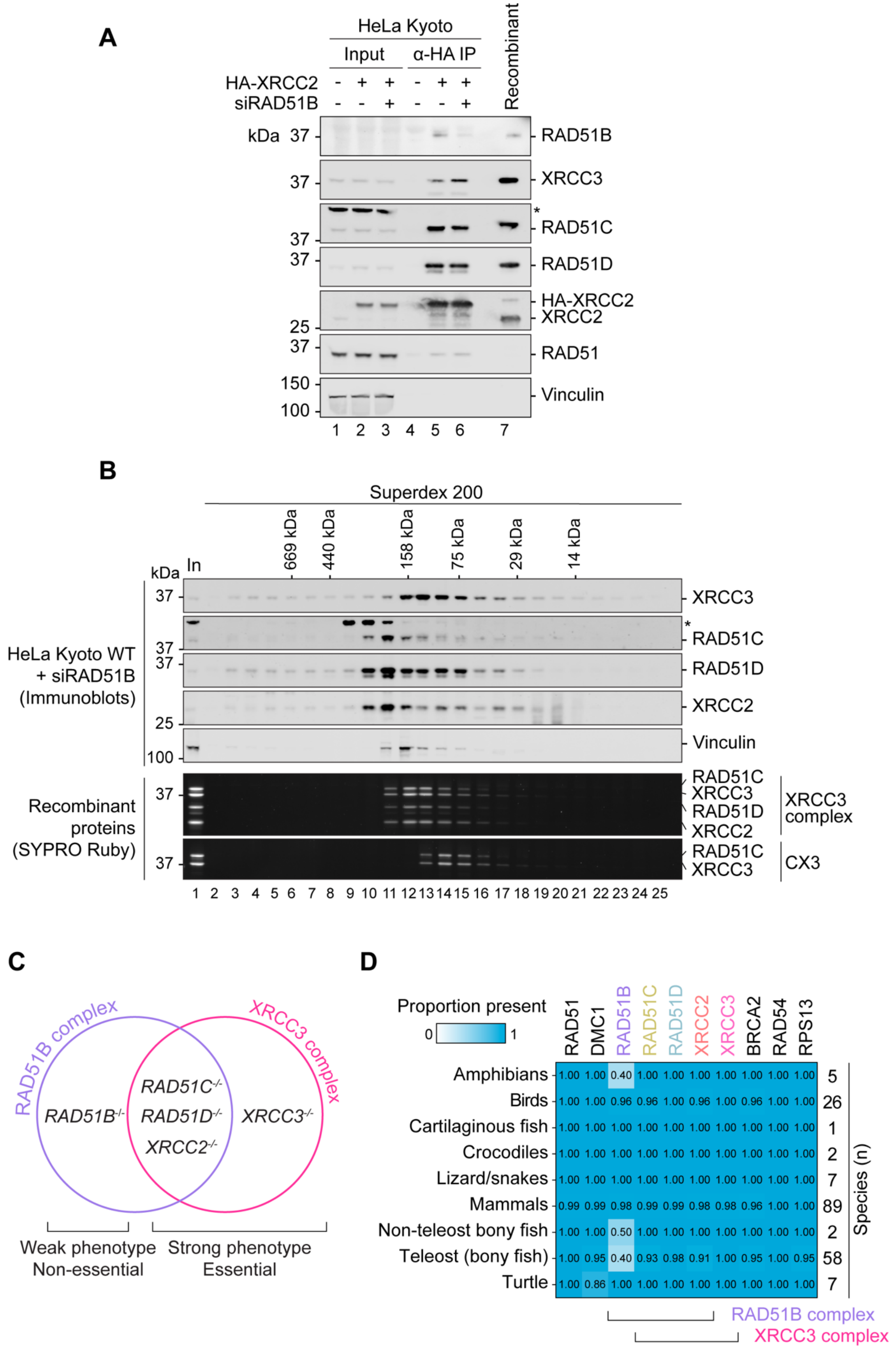
A
HeLa Kyoto
Input
α-HA IP
HA-XRCC2 - + + - + +
siRAD51B - - + - - +
Recombinant
kDa
37
150
100
25
RAD51B
XRCC3
*
RAD51C
RAD51D
HA-XRCC2
XRCC2
RAD51
Vinculin
1 2 3 4 5 6 7
B
Superdex 200
669 kDa
440 kDa
158 kDa
75 kDa
29 kDa
14 kDa
In
HeLa Kyoto WT + siRAD51B (Immunoblots)
Recombinant proteins (SYPRO Ruby)
XRCC3 complex
CX3
1 2 3 4 5 6 7 8 9 10 11 12 13 14 15 16 17 18 19 20 21 22 23 24 25
C
RAD51B complex
XRCC3 complex
RAD51B-/-
RAD51C-/-
RAD51D-/-
XRCC2-/-
XRCC3-/-
Weak phenotype
Non-essential
Strong phenotype
Essential
D
Proportion present
0
1
RAD51 DMC1 RAD51B RAD51C RAD51D XRCC2 XRCC3 BRCA2 RAD54 RPS13
Amphibians 1.00 1.00 0.40 1.00 1.00 1.00 1.00 1.00 1.00 1.00 5
Birds 1.00 1.00 0.96 0.96 1.00 0.96 1.00 0.96 1.00 1.00 26
Cartilaginous fish 1.00 1.00 1.00 1.00 1.00 1.00 1.00 1.00 1.00 1.00 1
Crocodiles 1.00 1.00 1.00 1.00 1.00 1.00 1.00 1.00 1.00 1.00 2
Lizard/snakes 1.00 1.00 1.00 1.00 1.00 1.00 1.00 1.00 1.00 1.00 7
Mammals 0.99 0.99 0.98 0.99 0.99 0.98 0.98 0.96 1.00 1.00 89
Non-teleost bony fish 1.00 1.00 0.50 1.00 1.00 1.00 1.00 1.00 1.00 1.00 2
Teleost (bony fish) 1.00 0.95 0.40 0.93 0.98 0.91 1.00 0.95 1.00 0.95 58
Turtle 1.00 0.86 1.00 1.00 1.00 1.00 1.00 1.00 1.00 1.00 7
Species (n)
RAD51B complex
XRCC3 complex

Fig. S1. Evidence for the heterotetrameric XRCC3 complex.

(A) Immunoprecipitation (α-HA) of RAD51 paralog complexes from HeLa Kyoto cell lines stably overexpressing HA-XRCC2 following siRNA-mediated depletion of RAD51B. Asterisk denotes non-specific band.

(B) Size-exclusion chromatography analysis of RAD51B-depleted HeLa Kyoto cell extracts compared with purified recombinant tetrameric XRCC3 and dimeric CX3 complexes. Asterisk denotes non-specific band.

(C) Venn diagram summarizing genetic data for the RAD51 paralog knockouts.

(D) Presence/absence matrix for RAD51, DMC1, RAD51B, RAD51C, RAD51D, XRCC2, XRCC3, BRCA2, RAD54 and RPS13 (ribosomal protein S13, used as a control) across multiple vertebrate classes. Numbers indicate proportion present, with number (n) of sampled species displayed on right.

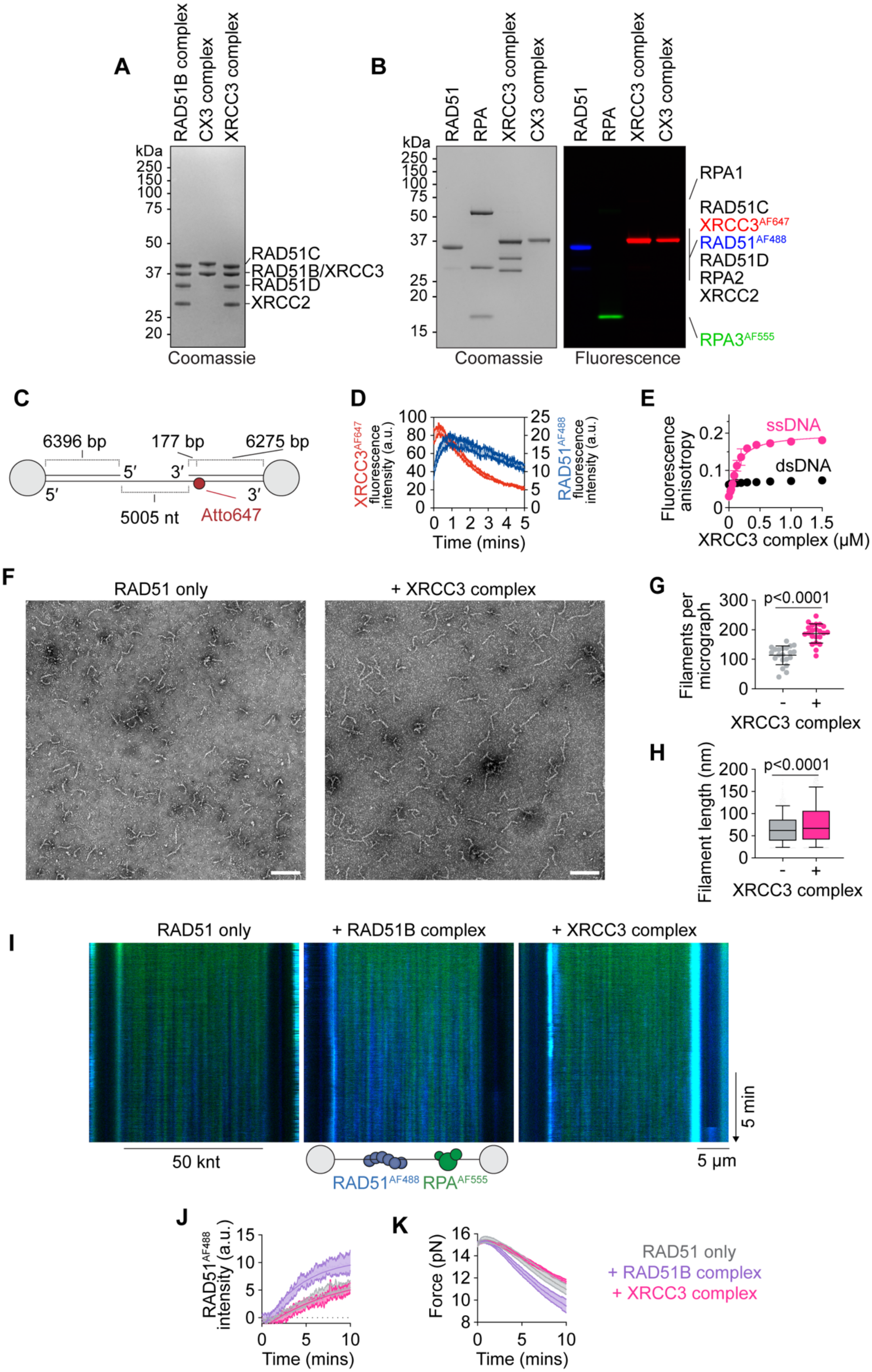

A
RAD51B complex
CX3 complex
XRCC3 complex
kDa
250
150
100
75
50
37
25
20
RAD51C
RAD51B/XRCC3
RAD51D
XRCC2
Coomassie
B
RAD51
RPA
XRCC3 complex
CX3 complex
RPA1
RAD51C
XRCC3AF647
RAD51AF488
RAD51D
RPA2
XRCC2
RPA3AF555
Coomassie
Fluorescence
C
6396 bp
177 bp
6275 bp
5005 nt
Atto647
D
XRCC3AF647 fluorescence intensity (a.u.)
RAD51AF488 fluorescence intensity (a.u.)
Time (mins)
E
Fluorescence anisotropy
ssDNA
dsDNA
XRCC3 complex (µM)
F
RAD51 only
+ XRCC3 complex
G
Filaments per micrograph
p<0.0001
XRCC3 complex
H
Filament length (nm)
p<0.0001
XRCC3 complex
I
RAD51 only
+ RAD51B complex
+ XRCC3 complex
5 min
50 knt
5 µm
RAD51AF488
RPAAF555
J
RAD51AF488 intensity (a.u.)
Time (mins)
K
Force (pN)
Time (mins)
RAD51 only
+ RAD51B complex
+ XRCC3 complex

Fig. S2. Purification and biochemical functions of the XRCC3 complex.

(A) SDS-PAGE of RAD51 paralog complexes purified from baculovirus-infected insect cells.

(B) SDS-PAGE of fluorescent proteins used in the study: RAD51^{AF488}, RPA^{AF555}, XRCC3^{AF647} and CX3^{AF647} complex.

(C) Schematic illustration of gapped DNA substrate used for the single molecule experiments of Fig. 1C.

(D) Fluorescence intensities of the XRCC3^{AF647} complex and RAD51^{AF488} signal over time. n=6 independent ssDNA molecules. Shaded area = SEM. a.u. = arbitrary units.

(E) Fluorescence anisotropy measurements of ssDNA (n=10 independent experiments) and dsDNA (n=3 independent experiments) binding by the XRCC3 complex. Data are mean ± SD.

(F) Representative negative stain electron microscopy micrographs of RAD51-ssDNA nucleoprotein filaments formed in the absence and presence of XRCC3 complex. Scale bar, 100 nm.

(G) Scatter plot showing the number of RAD51 filaments per micrograph in the absence (n=21 micrographs) and presence (n=22 micrographs) of XRCC3 complex (black lines = mean + S.D). Unpaired t-test.

(H) Box plot of RAD51 filament lengths formed in the absence (n=2,389 filaments) and presence (n=4,127 filaments) of the XRCC3 complex. Centre line, median; box bounds, interquartile range; whiskers, 5th and 95th percentiles. Mann-Whitney U test.

(I) Representative kymographs showing the assembly of RAD51^{AF488} (blue) on RPA^{AF555} (green) coated λ-ssDNA in the absence (left) and presence of RAD51B (middle) or XRCC3 (right) complexes. Bottom, schematic illustration of the Lumick's C-trap experiment. a.u. = arbitrary units.

(J) Normalized fluorescence intensity of RAD51^{AF488} signal over time in the absence of RAD51 paralogs (n=6 independent ssDNA molecules), with the RAD51B complex (n=6) and with the XRCC3 complex (n=5 independent ssDNA molecules). n values represent the number of independent ssDNA molecules. Shaded area represents SEM. Solid line represents exponential curve fit.

(K) Force measurement between traps over time in the absence of paralogs (n=7 molecules), with RAD51B complex (n=7) and with XRCC3 complex (n=6). Shaded bars represent SEM, and the solid curve represents the mean. n values represent the number of independent ssDNA molecules.

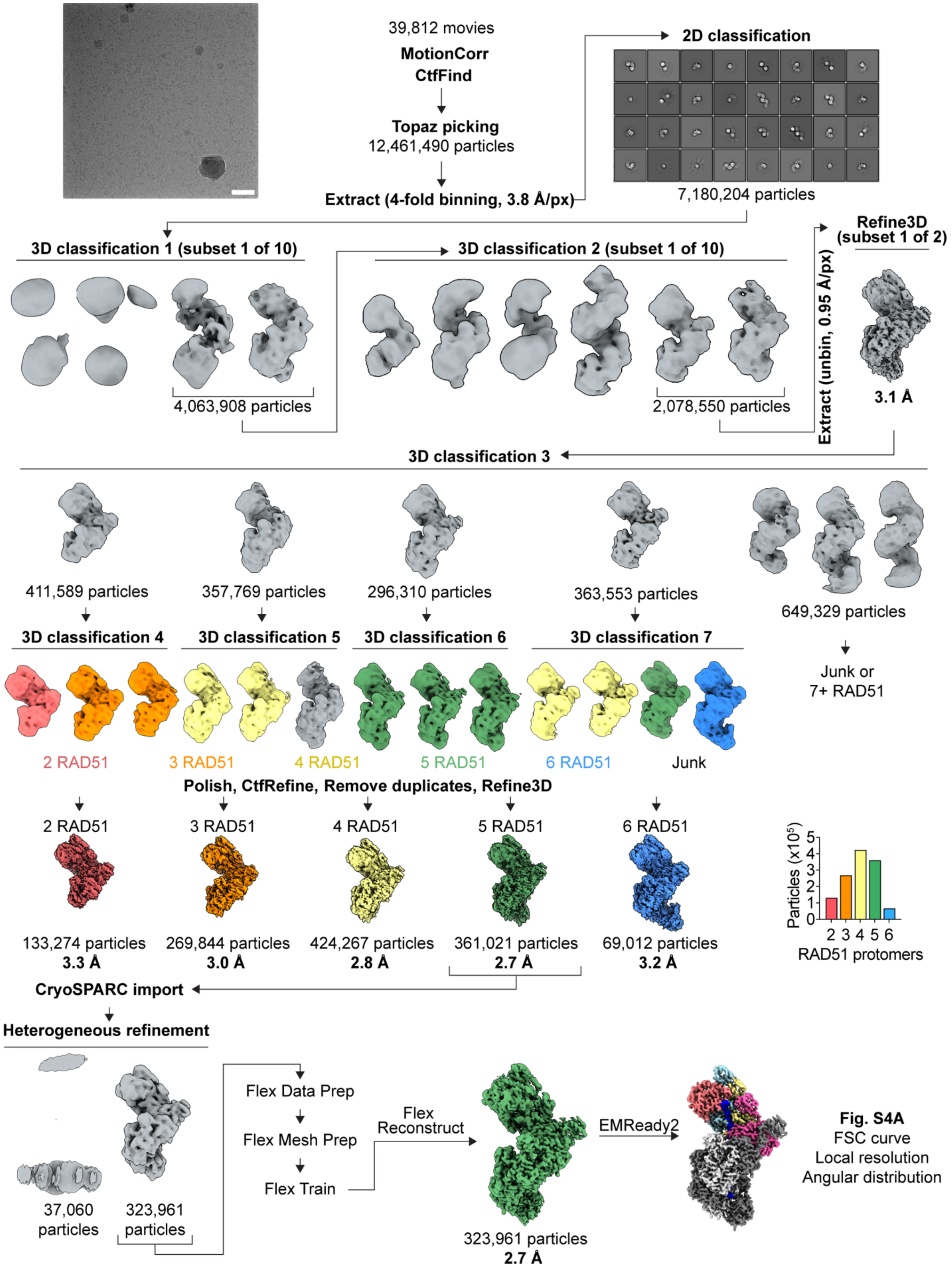
39,812 movies
MotionCorr
CtfFind
Topaz picking
12,461,490 particles
Extract (4-fold binning, 3.8 Å/px)
2D classification
7,180,204 particles
3D classification 1 (subset 1 of 10)
4,063,908 particles
3D classification 2 (subset 1 of 10)
2,078,550 particles
Extract (unbin, 0.95 Å/px)
Refine3D (subset 1 of 2)
3.1 Å
3D classification 3
411,589 particles
357,769 particles
296,310 particles
363,553 particles
649,329 particles
Junk or 7+ RAD51
3D classification 4
3D classification 5
3D classification 6
3D classification 7
2 RAD51
3 RAD51
4 RAD51
5 RAD51
6 RAD51
Junk
Polish, CtfRefine, Remove duplicates, Refine3D
2 RAD51
133,274 particles
3.3 Å
3 RAD51
269,844 particles
3.0 Å
4 RAD51
424,267 particles
2.8 Å
5 RAD51
361,021 particles
2.7 Å
6 RAD51
69,012 particles
3.2 Å
Particles (x10⁵)
RAD51 protomers
CryoSPARC import
Heterogeneous refinement
37,060 particles
323,961 particles
Flex Data Prep
Flex Mesh Prep
Flex Train
Flex Reconstruct
323,961 particles
2.7 Å
EMReady2
Fig. S4A
FSC curve
Local resolution
Angular distribution

Fig. S3. Single particle analysis pipeline of XRCC3-RAD51C-RAD51D-XRCC2 capped RAD51-ssDNA filament.
Micrograph scale bar = 50 nm.

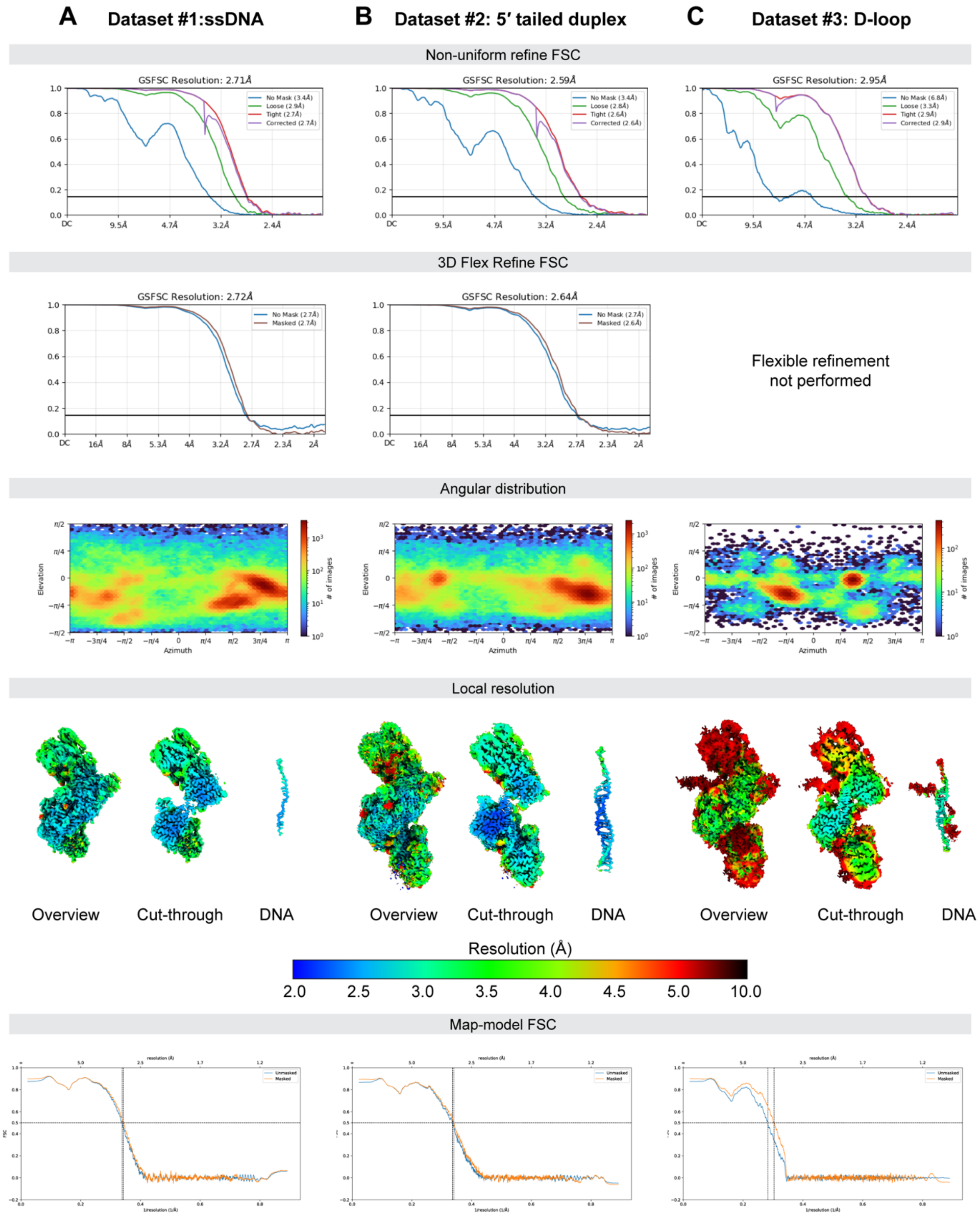
A
Dataset #1:ssDNA
B
Dataset #2: 5′ tailed duplex
C
Dataset #3: D-loop
Non-uniform refine FSC
GSFSC Resolution: 2.71Å
No Mask (3.4Å)
Loose (2.9Å)
Tight (2.7Å)
Corrected (2.7Å)
GSFSC Resolution: 2.59Å
No Mask (3.4Å)
Loose (2.8Å)
Tight (2.6Å)
Corrected (2.6Å)
GSFSC Resolution: 2.95Å
No Mask (6.8Å)
Loose (3.3Å)
Tight (2.9Å)
Corrected (2.9Å)
3D Flex Refine FSC
GSFSC Resolution: 2.72Å
No Mask (2.7Å)
Masked (2.7Å)
GSFSC Resolution: 2.64Å
No Mask (2.7Å)
Masked (2.6Å)
Flexible refinement not performed
Angular distribution
Elevation
Azimuth
# of images
Local resolution
Overview
Cut-through
DNA
Overview
Cut-through
DNA
Overview
Cut-through
DNA
Resolution (Å)
2.0
2.5
3.0
3.5
4.0
4.5
5.0
10.0
Map-model FSC
Unmasked
Masked

Fig. S4. FSC curves, angular distribution and local resolution of cryo-EM maps.

(A) XRCC3-RAD51C-RAD51D-XRCC2 capped RAD51-ssDNA filament (analysis pipeline, fig. S3).
(B) XRCC3-RAD51C-RAD51D-XRCC2 capped RAD51 tailed duplex filament (analysis pipeline, fig. S8).
(C) XRCC3-RAD51C-RAD51D-XRCC2 capped RAD51 D-loop (analysis pipeline, fig. S11).

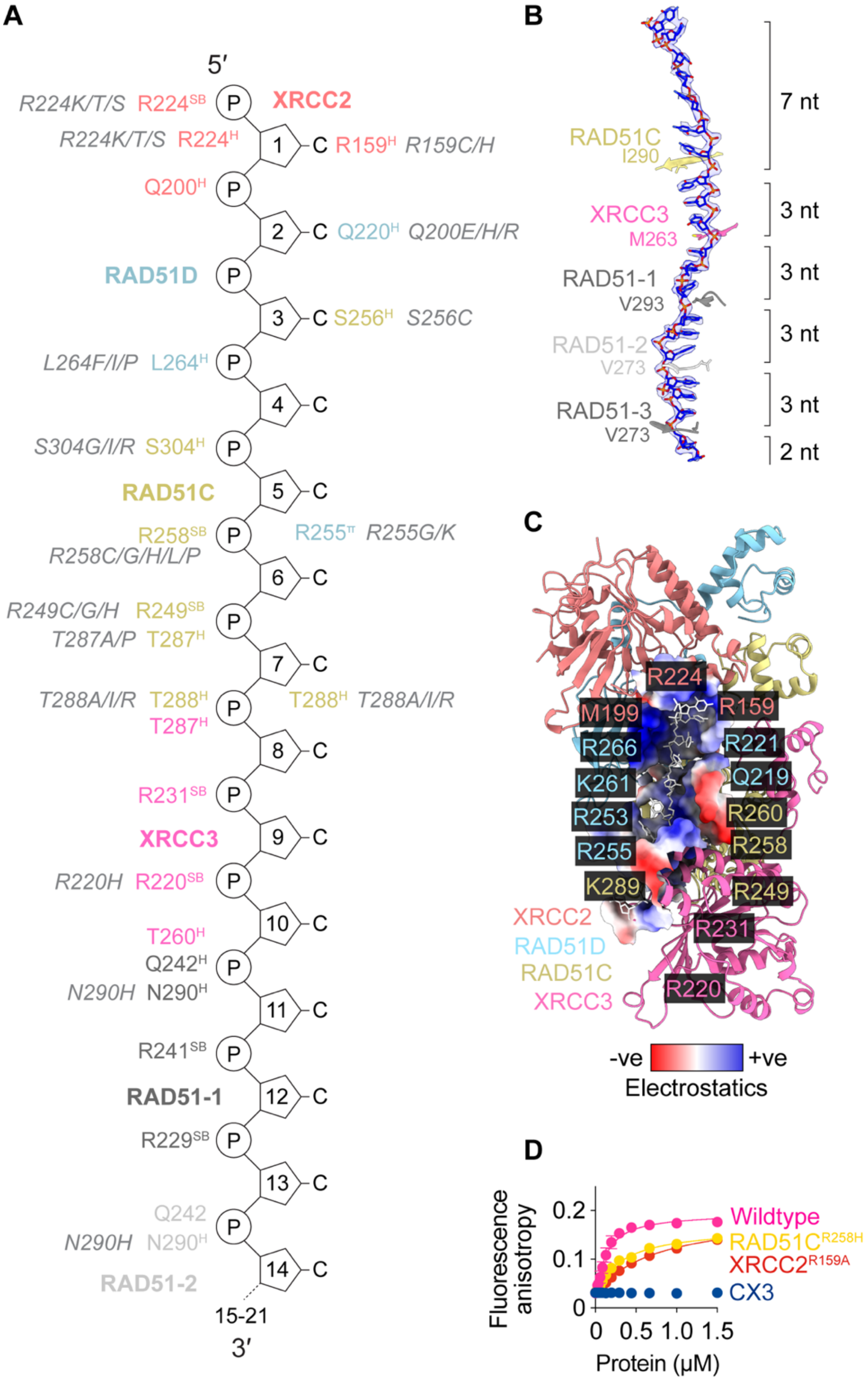
A
5′
R224K/T/S R224SB
XRCC2
R224K/T/S R224H
R159H R159C/H
Q200H
Q220H Q200E/H/R
RAD51D
S256H S256C
L264F/I/P L264H
S304G/I/R S304H
RAD51C
R258SB
R258C/G/H/L/P
R255π R255G/K
R249C/G/H R249SB
T287A/P T287H
T288A/I/R T288H
T287H
T288H T288A/I/R
R231SB
XRCC3
R220H R220SB
T260H
Q242H
N290H N290H
R241SB
RAD51-1
R229SB
Q242
N290H N290H
RAD51-2
15-21
3′
B
7 nt
RAD51C
I290
3 nt
XRCC3
M263
3 nt
RAD51-1
V293
3 nt
RAD51-2
V273
3 nt
RAD51-3
V273
2 nt
C
R224
M199
R159
R266
R221
K261
Q219
R253
R260
R255
R258
K289
R249
R231
R220
XRCC2
RAD51D
RAD51C
XRCC3
-ve
+ve
Electrostatics
D
Fluorescence anisotropy
0.2
0.1
0
0 0.5 1.0 1.5
Protein (μM)
Wildtype
RAD51CR258H
XRCC2R159A
CX3

Fig. S5. Interactions of RAD51, XRCC3, RAD51C, RAD51D and XRCC2 with ssDNA.

(A) 2D plot of ssDNA (nucleotides 1-14) highlighting key residues interacting with phosphates, sugars and cytosine bases and associated missense variants (italicized) identified in the ClinVar database. RAD51 binds to nucleotides 15-21 in a manner analogous to nucleotides 12, 13 and 14. Superscript H, SB and π indicate hydrogen bonding, salt bridge and cation-π interactions, respectively.

(B) Cryo-EM density and atomic model of ssDNA, showing triplet stacking pattern mediated by hydrophobic residues $RAD51^{V273}$, $XRCC3^{M263}$ and $RAD51C^{I290}$.

(C) Atomic model of XRCC3 complex with surface representation of electrostatic residues. ssDNA represented in white.

(D) Fluorescence anisotropy measurements of ssDNA binding by wildtype (n=10) and mutant ($RAD51C^{R258H}$ [n=3], $XRCC2^{R159A}$ [n=3]) XRCC3 complex, and CX3 (n=3). Data are mean ± SD. n refers to independent experiments.

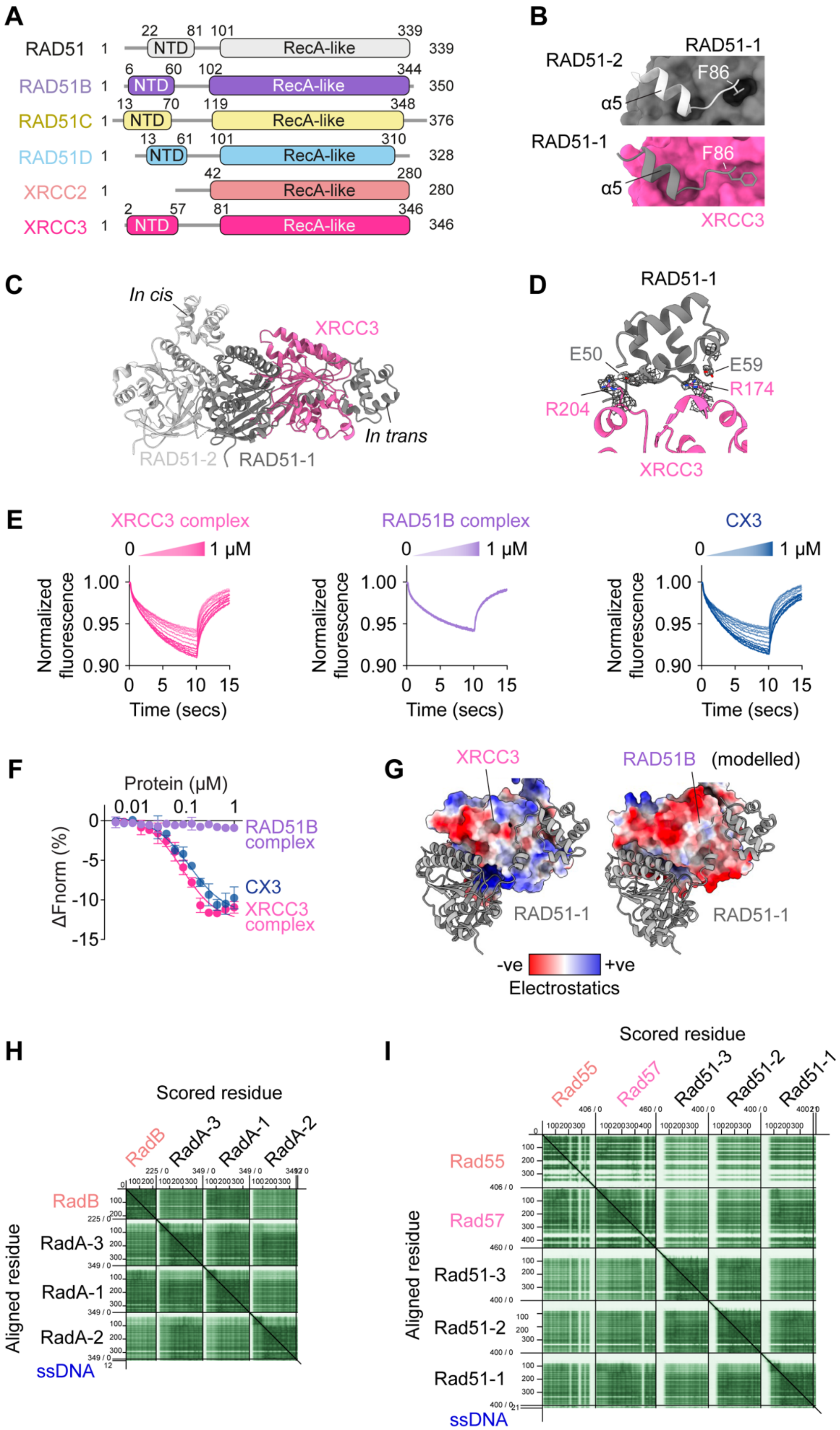

A
RAD51
RAD51B
RAD51C
RAD51D
XRCC2
XRCC3
NTD
RecA-like
B
RAD51-2
RAD51-1
F86
α5
XRCC3
C
In cis
In trans
XRCC3
RAD51-2
RAD51-1
D
RAD51-1
E50
E59
R174
R204
XRCC3
E
XRCC3 complex
RAD51B complex
CX3
0
1 μM
Normalized fluorescence
Time (secs)
F
Protein (μM)
ΔFnorm (%)
RAD51B complex
CX3
XRCC3 complex
G
XRCC3
RAD51B
(modelled)
RAD51-1
-ve
+ve
Electrostatics
H
Scored residue
Aligned residue
RadB
RadA-3
RadA-1
RadA-2
ssDNA
I
Scored residue
Aligned residue
Rad55
Rad57
Rad51-3
Rad51-2
Rad51-1
ssDNA

Fig. S6. RAD51^{NTD} remodeling upon binding XRCC3 and evolutionary conservation.

(A) Domain architectures of RAD51 and the RAD51 paralogs.

(B) RAD51 oligomerization is driven by RAD51^{F86} binding a hydrophobic pocket, which is absent in XRCC3.

(C) Atomic model of XRCC3^{CTD}, RAD51-1 and RAD51-2 showing the *in cis* arrangement of RAD51-2^{NTD} and *in trans* arrangement of XRCC3-interacting RAD51-1^{NTD}.

(D) Atomic model of RAD51^{NTD} interacting with XRCC3^{CTD}. Two salt bridges form between RAD51^{E50}:XRCC3^{R204} and RAD51^{E59}:XRCC3^{R174}. Cryo-EM map of interacting residues shown by black mesh.

(E) Representative raw microscale thermophoresis (MST) traces of XRCC3 complex (left), RAD51B complex (middle) and CX3 heterodimer (right) binding to RAD51^{AF488} in the presence of 1 mM ATP and $CaCl_2$.

(F) MST dose response curves of XRCC3 complex (n=3), RAD51B complex (n=3) and CX3 (n=3) binding to RAD51^{AF488}. XRCC3 complex K_D = 57 nM [34-95 nM, 95% CI]. RAD51B complex = no binding. CX3 complex K_D = 93 nM [60-143, 95% CI]. Data are mean ± SD. n refers to independent experiments.

(G) Atomic model of RAD51 and electrostatic surface representation of XRCC3^{CTD} showing that *in trans* rotation of RAD51^{NTD} is driven by its attraction to an electropositive patch. An equivalent electropositive patch is absent in the modeled RAD51B^{CTD}, prohibiting its interaction.

(H) AlphaFold3 PAE plots for the *Pyrococcus furiosus* RadB capped RadA-ssDNA filaments.

(I) AlphaFold3 PAE plots for the *Saccharomyces cerevisiae* Rad55-Rad57 capped Rad51-ssDNA filaments.

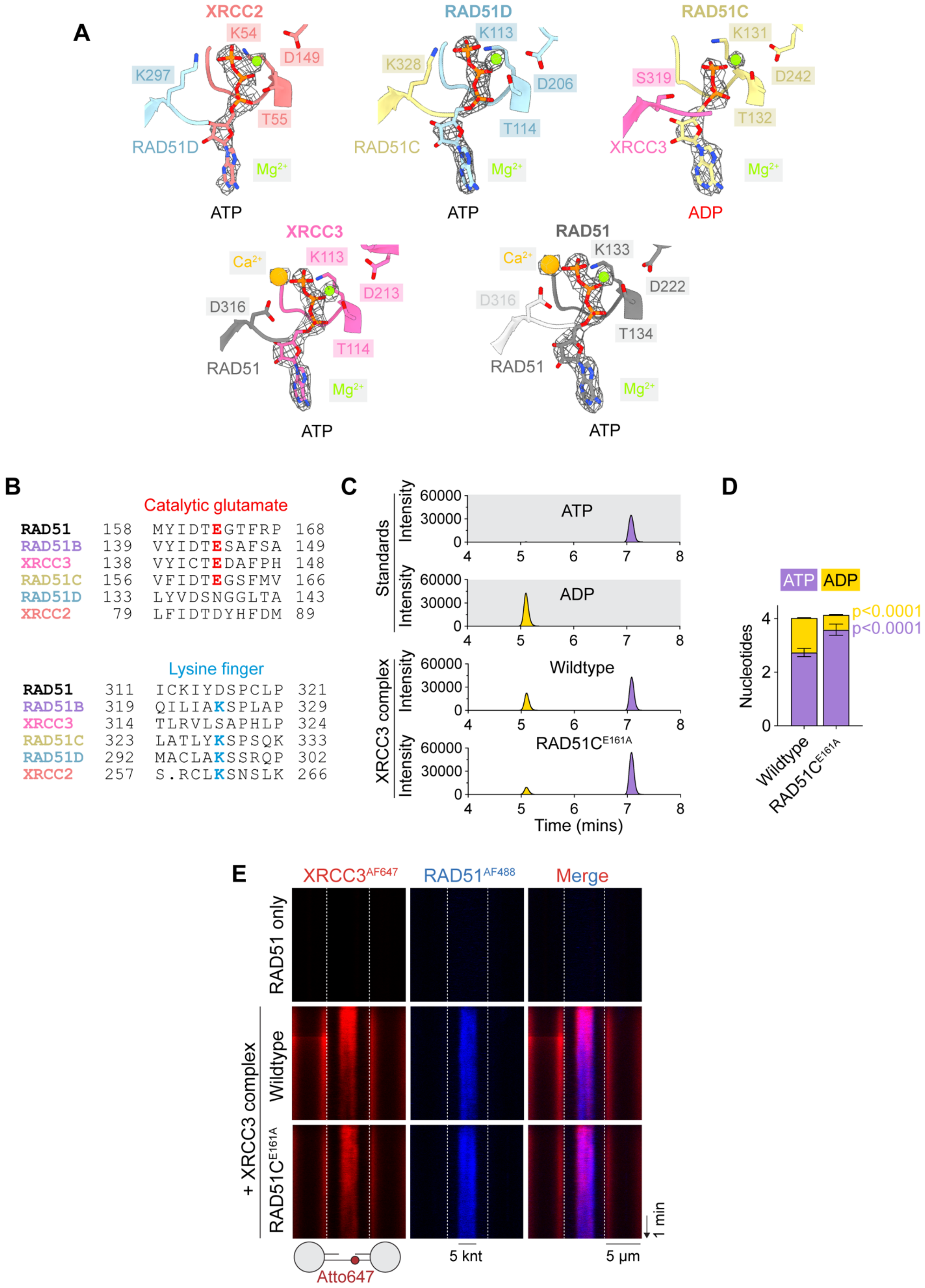

A
XRCC2
K54
D149
K297
T55
RAD51D
Mg2+
ATP
RAD51D
K113
K328
D206
T114
RAD51C
Mg2+
ATP
RAD51C
K131
D242
S319
T132
XRCC3
Mg2+
ADP
XRCC3
Ca2+
K113
D213
D316
T114
RAD51
Mg2+
ATP
RAD51
Ca2+
K133
D222
D316
T134
RAD51
Mg2+
ATP
B
Catalytic glutamate
RAD51 158 MYIDTEGTFRP 168
RAD51B 139 VYIDTESAFSA 149
XRCC3 138 VYICTEDAFPH 148
RAD51C 156 VFIDTEGSFMV 166
RAD51D 133 LYVDSNGGLTA 143
XRCC2 79 LFIDTDYHFDM 89
Lysine finger
RAD51 311 ICKIYDSPCLP 321
RAD51B 319 QILIAKSPLAP 329
XRCC3 314 TLRVLSAPHLP 324
RAD51C 323 LATLYKSPSQK 333
RAD51D 292 MACLAKSSRQP 302
XRCC2 257 S.RCLKSNSLK 266
C
Standards
XRCC3 complex
Intensity
ATP
ADP
Wildtype
RAD51CE161A
Time (mins)
D
ATP
ADP
Nucleotides
p<0.0001
p<0.0001
Wildtype
RAD51CE161A
E
XRCC3AF647
RAD51AF488
Merge
RAD51 only
+ XRCC3 complex
Wildtype
RAD51CE161A
1 min
5 knt
5 µm
Atto647

Fig. S7. Nucleotide binding and hydrolysis by the XRCC3 complex.

(A) Molecules of ATP bound to XRCC2, RAD51D, XRCC3 and RAD51, and ADP bound to RAD51C. Residues shown include the Walker A lysine and threonine, and Walker B aspartate. Lysine residues (RAD51D^{K297} and RAD51C^{K328}) transcoordinate the γ-phosphate of ATP bound to XRCC2 and RAD51D. A serine (XRCC3^{S319}) occupies an equivalent position in the RAD51C active site. Aspartate (RAD51^{D316}) coordinates the calcium ion bound in the RAD51 and XRCC3 active sites. Cryo-EM density of nucleotide, magnesium (green) and calcium (orange) ions shown as black mesh.

(B) Multiple sequence alignments of RAD51, RAD51B, RAD51C, RAD51D, XRCC2 and XRCC3 showing the catalytic glutamate residues (asparagine in RAD51D, aspartate in XRCC2) and lysine finger residues (aspartate in RAD51, serine in XRCC3).

(C) High performance liquid chromatograph chromatograms of ATP and ADP nucleotide standards, and nucleotides extracted from wildtype and RAD51C^{E161A} mutant XRCC3 complexes.

(D) Stacked bar chart (mean ± SD) quantifying number of nucleotides bound by wildtype (n=5 technical measurements across two experiments) and RAD51C^{E161A} mutant (n=5 technical measurements across two experiments) XRCC3 complexes.

(E) Representative kymographs showing the selective binding of RAD51^{AF488} (blue) to ssDNA in the absence and presence of wildtype and RAD51C^{E161A} mutant XRCC3^{AF647} complexes (red). The gapped DNA substrate is indicated below. White dashed lines indicate bead-DNA boundaries.

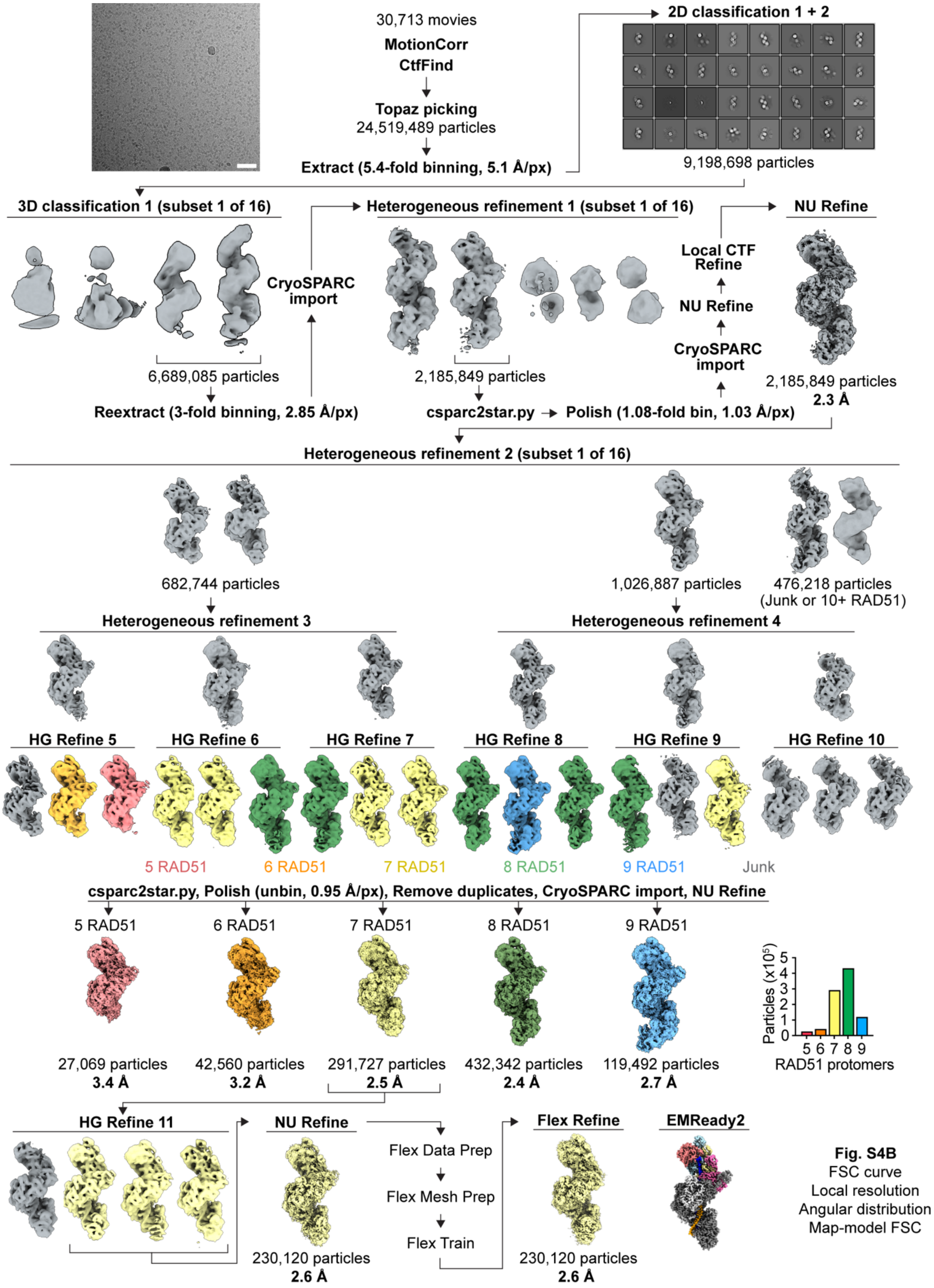
30,713 movies
MotionCorr
CtfFind
Topaz picking
24,519,489 particles
Extract (5.4-fold binning, 5.1 Å/px)
2D classification 1 + 2
9,198,698 particles
3D classification 1 (subset 1 of 16)
6,689,085 particles
Reextract (3-fold binning, 2.85 Å/px)
CryoSPARC import
Heterogeneous refinement 1 (subset 1 of 16)
2,185,849 particles
csparc2star.py
Polish (1.08-fold bin, 1.03 Å/px)
CryoSPARC import
NU Refine
Local CTF Refine
NU Refine
2,185,849 particles
2.3 Å
Heterogeneous refinement 2 (subset 1 of 16)
682,744 particles
1,026,887 particles
476,218 particles
(Junk or 10+ RAD51)
Heterogeneous refinement 3
Heterogeneous refinement 4
HG Refine 5
HG Refine 6
HG Refine 7
HG Refine 8
HG Refine 9
HG Refine 10
5 RAD51
6 RAD51
7 RAD51
8 RAD51
9 RAD51
Junk
csparc2star.py, Polish (unbin, 0.95 Å/px), Remove duplicates, CryoSPARC import, NU Refine
5 RAD51
6 RAD51
7 RAD51
8 RAD51
9 RAD51
27,069 particles
3.4 Å
42,560 particles
3.2 Å
291,727 particles
2.5 Å
432,342 particles
2.4 Å
119,492 particles
2.7 Å
Particles (x10^5)
0 1 2 3 4 5
5 6 7 8 9
RAD51 protomers
HG Refine 11
NU Refine
230,120 particles
2.6 Å
Flex Data Prep
Flex Mesh Prep
Flex Train
Flex Refine
230,120 particles
2.6 Å
EMReady2
Fig. S4B
FSC curve
Local resolution
Angular distribution
Map-model FSC

Fig. S8. Single particle analysis pipeline of the XRCC3-RAD51C-RAD51D-XRCC2 capped RAD51 on a partial duplex substrate.
Micrograph scale bar = 50 nm.

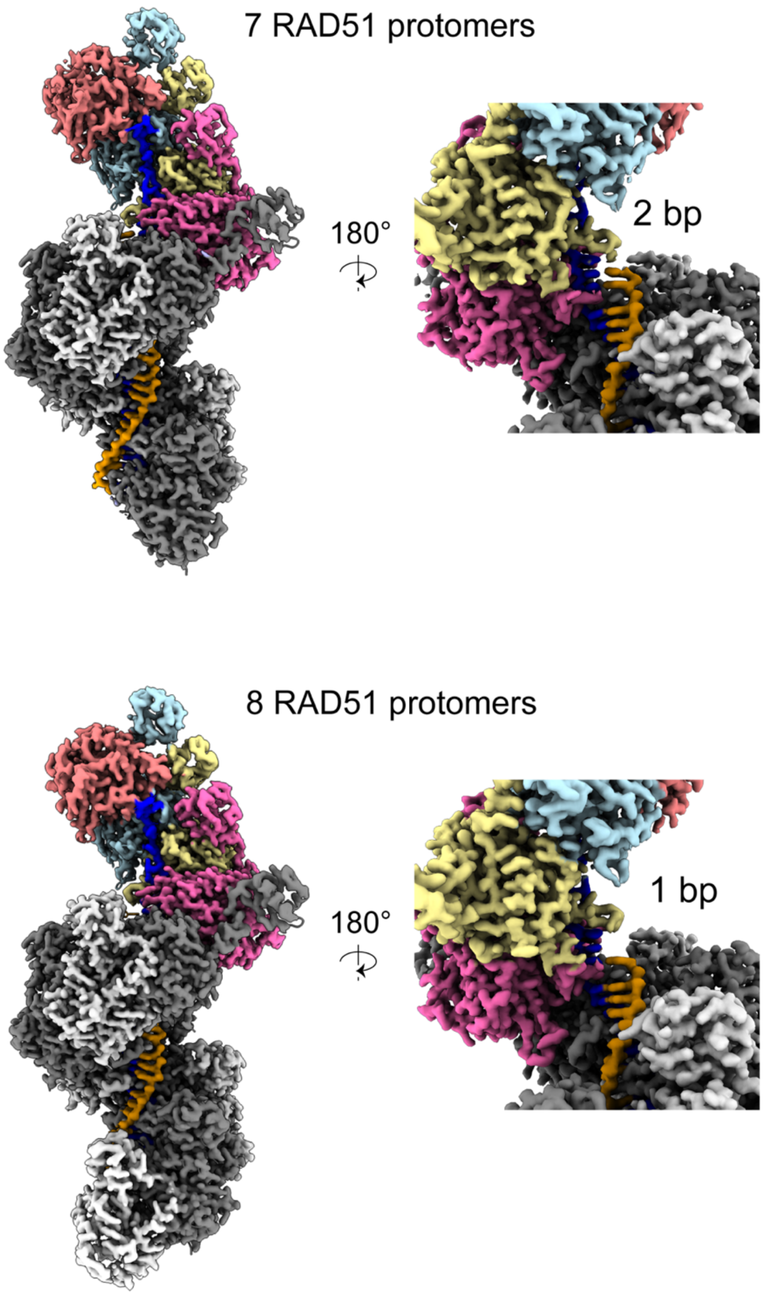
7 RAD51 protomers
180°
2 bp
8 RAD51 protomers
180°
1 bp

Fig. S9. ssDNA-dsDNA binding by the XRCC3 complex does not set duplex registry.
Comparison of seven- and eight-RAD51 protomer tailed duplex filaments, capped by the XRCC3 complex, with the XRCC3-bound triplet pairing one or two complementary bases, respectively. The interaction does not set the binding register, as eight-protomer filaments pair only one nucleotide within the XRCC3 triplet, whereas a junction-defined register would yield two in all cases.

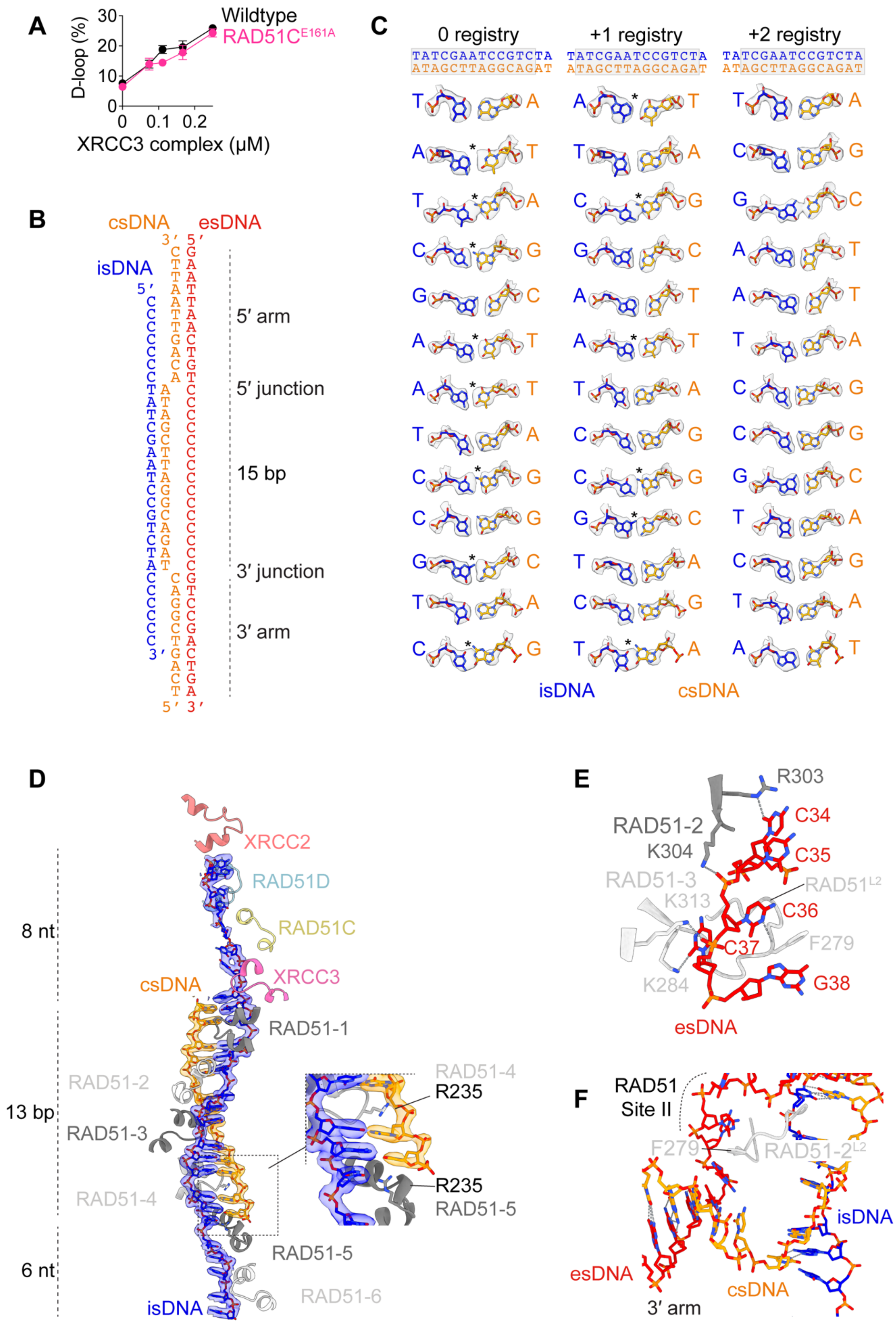
A
D-loop (%)
XRCC3 complex (μM)
Wildtype
RAD51C E161A
B
csDNA
esDNA
isDNA
5′ arm
5′ junction
15 bp
3′ junction
3′ arm
C
0 registry
+1 registry
+2 registry
TATCGAATCCGTCTA
ATAGCTTAGGCAGAT
isDNA
csDNA
D
XRCC2
RAD51D
RAD51C
XRCC3
csDNA
RAD51-1
RAD51-2
RAD51-3
RAD51-4
RAD51-5
RAD51-6
isDNA
8 nt
13 bp
6 nt
R235
E
R303
C34
C35
C36
C37
G38
RAD51-2
K304
RAD51-3
K313
RAD51 L2
F279
K284
esDNA
F
RAD51 Site II
F279
RAD51-2 L2
isDNA
esDNA
csDNA
3′ arm

Fig. S10. XRCC3 complex caps a RAD51 D-loop.

(A) Quantification of RAD51 D-loop stimulation by wildtype (n=3) and RAD51C^{E161A} (n=3) XRCC3 complexes. Data are mean ± SD. n refers to independent experiments.

(B) Sequences of the invading strand DNA (isDNA), complementary strand DNA (csDNA) and exchanged strand DNA (esDNA). 15-bp isDNA:csDNA proposed base pairing is highlighted. 5′ and 3′ duplex arms, and 5′ and 3′ junctions, are defined 5′ to 3′ relative to the isDNA. Full sequences are detailed in materials and methods.

(C) Registry analysis of base pairing in the D-loop. Comparison of nucleotide fitting into cryo-EM density supports a +2 registry for the paired dsDNA, relative to 0 and +1 registry models. Map at 9σ contour level.

(D) Atomic model and cryo-EM density of isDNA and the csDNA duplex. XRCC3, RAD51C, RAD51D, XRCC2 and RAD51 L1 loops are shown in cartoon representation. Inset shows RAD51^{R235} π-cation stabilization of triplets and the unpaired nucleotide in the RAD51-5 triplet.

(E) Atomic model highlighting esDNA bases C34, C35, C36, C37, and G38. RAD51 residue F279 stacks against G38, while residues K284, R303, K304, and K313 interact with the phosphate backbone and bases of the esDNA strand.

(F) Atomic model of the 3′ junction, with RAD51^{F279} stacking against the 3′ duplex arm.

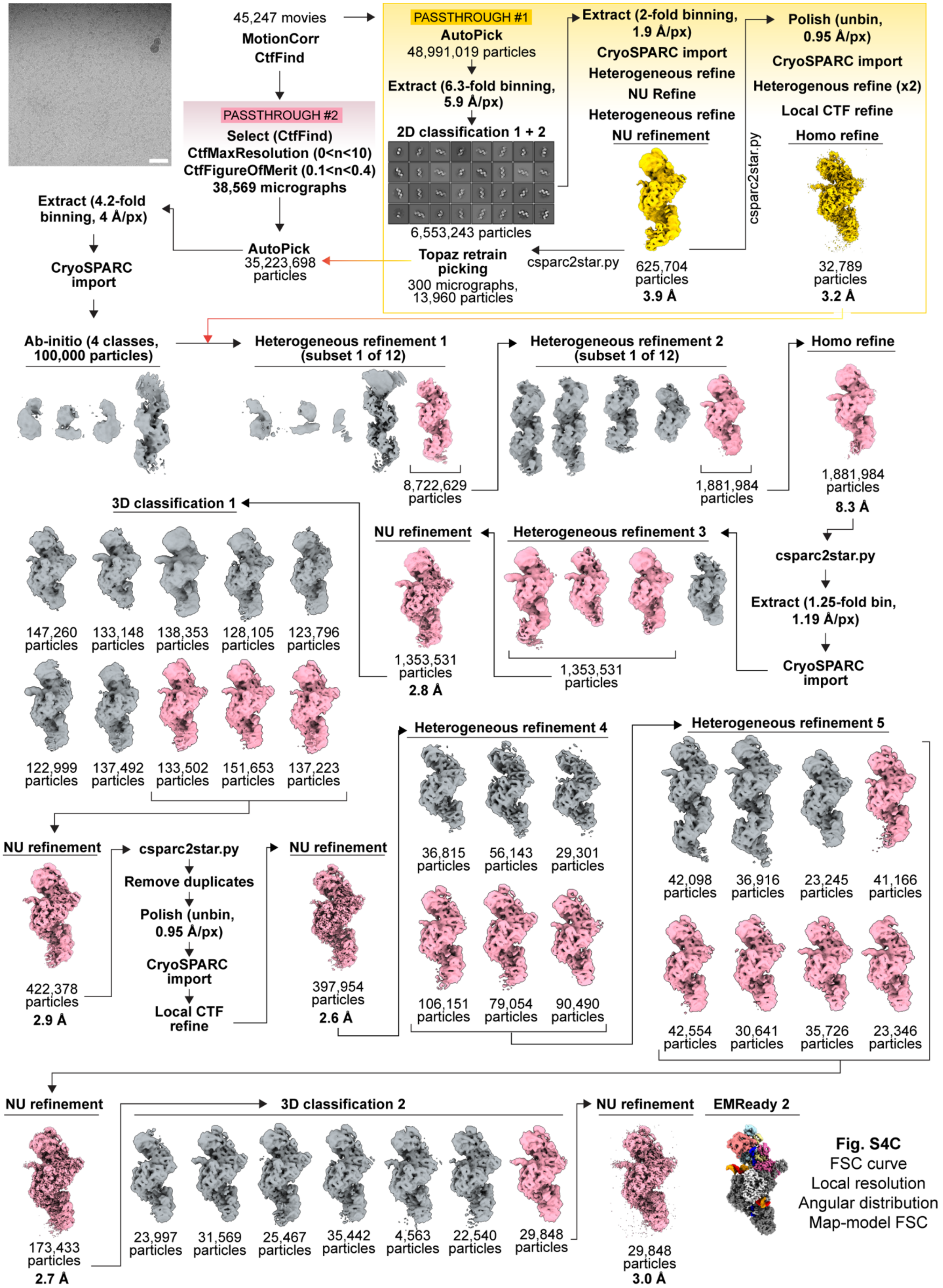
45,247 movies
MotionCorr
CtfFind
PASSTHROUGH #2
Select (CtfFind)
CtfMaxResolution (0<n<10)
CtfFigureOfMerit (0.1<n<0.4)
38,569 micrographs
PASSTHROUGH #1
AutoPick
48,991,019 particles
Extract (6.3-fold binning, 5.9 Å/px)
2D classification 1 + 2
6,553,243 particles
Extract (2-fold binning, 1.9 Å/px)
CryoSPARC import
Heterogeneous refine
NU Refine
Heterogeneous refine
NU refinement
625,704 particles
3.9 Å
csparc2star.py
Polish (unbin, 0.95 Å/px)
CryoSPARC import
Heterogenous refine (x2)
Local CTF refine
Homo refine
32,789 particles
3.2 Å
csparc2star.py
Topaz retrain picking
300 micrographs, 13,960 particles
AutoPick
35,223,698 particles
Extract (4.2-fold binning, 4 Å/px)
CryoSPARC import
Ab-initio (4 classes, 100,000 particles)
Heterogeneous refinement 1 (subset 1 of 12)
8,722,629 particles
Heterogeneous refinement 2 (subset 1 of 12)
1,881,984 particles
Homo refine
1,881,984 particles
8.3 Å
csparc2star.py
Extract (1.25-fold bin, 1.19 Å/px)
CryoSPARC import
Heterogeneous refinement 3
1,353,531 particles
NU refinement
1,353,531 particles
2.8 Å
3D classification 1
147,260 particles
133,148 particles
138,353 particles
128,105 particles
123,796 particles
122,999 particles
137,492 particles
133,502 particles
151,653 particles
137,223 particles
NU refinement
422,378 particles
2.9 Å
csparc2star.py
Remove duplicates
Polish (unbin, 0.95 Å/px)
CryoSPARC import
Local CTF refine
NU refinement
397,954 particles
2.6 Å
Heterogeneous refinement 4
36,815 particles
56,143 particles
29,301 particles
106,151 particles
79,054 particles
90,490 particles
Heterogeneous refinement 5
42,098 particles
36,916 particles
23,245 particles
41,166 particles
42,554 particles
30,641 particles
35,726 particles
23,346 particles
NU refinement
173,433 particles
2.7 Å
3D classification 2
23,997 particles
31,569 particles
25,467 particles
35,442 particles
4,563 particles
22,540 particles
29,848 particles
NU refinement
29,848 particles
3.0 Å
EMReady 2
Fig. S4C
FSC curve
Local resolution
Angular distribution
Map-model FSC

Fig. S11. Single particle analysis pipeline of the XRCC3-RAD51C-RAD51D-XRCC2 capped RAD51 on a D-loop intermediate.
Micrograph scale bar = 50 nm.

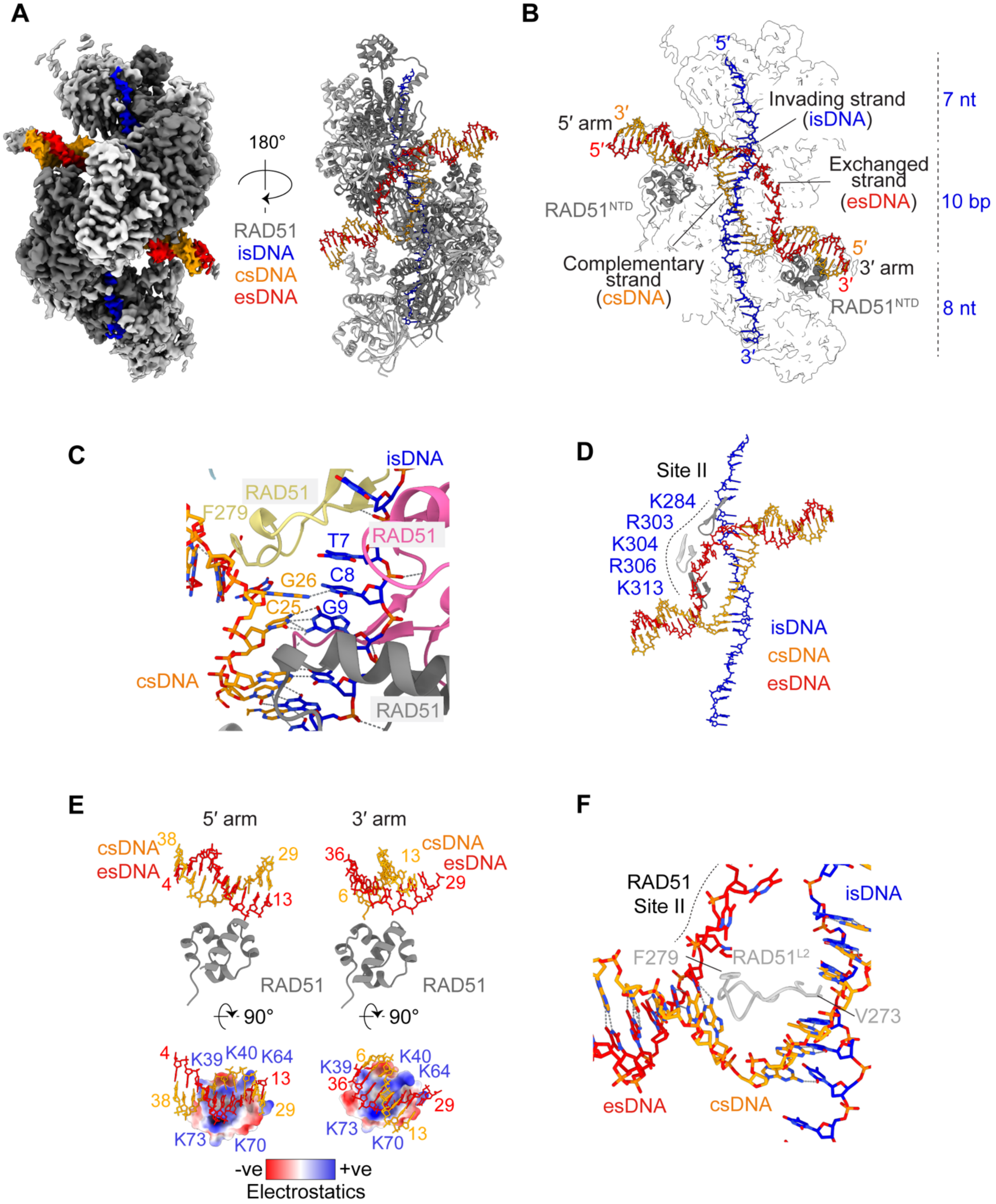
A
180°
RAD51
isDNA
csDNA
esDNA
B
5′
3′
5′ arm
5′
Invading strand
(isDNA)
Exchanged
strand
(esDNA)
RAD51NTD
Complementary
strand
(csDNA)
5′
3′ arm
3′
RAD51NTD
3′
7 nt
10 bp
8 nt
C
isDNA
RAD51
F279
RAD51
T7
C8
G26
C25
G9
csDNA
RAD51
D
Site II
K284
R303
K304
R306
K313
isDNA
csDNA
esDNA
E
5′ arm
3′ arm
csDNA
38
29
esDNA
4
13
36
13
csDNA
esDNA
29
6
RAD51
RAD51
90°
90°
4
K39
K40
K64
13
38
29
K73
K70
6
K40
K39
K64
36
29
K73
K70
13
-ve
+ve
Electrostatics
F
RAD51
Site II
isDNA
F279
RAD51L2
V273
esDNA
csDNA

Fig. S12. RAD51-only D-loop, for comparison with the XRCC3 complex-capped RAD51 D-loop.
(A) Cryo-EM structure of the 10-bp RAD51-only D-loop. Left: cryo-EM density map (EMDB-52646). Right: atomic model (PDB-9I62) rotated 180°.
(B) Reference map outlining the cryo-EM density and atomic model of invading strand DNA (isDNA), complementary strand DNA (csDNA), and exchanged strand DNA (esDNA). Key features of interest include 5′/3′ arms, and duplex-bound RAD51 NTDs.
(C) Atomic model of the 5′ junction, colored as in Fig. 6F to facilitate direct comparison with the XRCC3-capped structure.
(D) Atomic model highlighting DNA binding site II (K284, R303, K304, R306, K313), which engages the esDNA strand.
(E) Top, atomic model of $RAD51^{NTD}$s and their associated 5′ and 3′ duplex DNA arms. Bottom, electrostatic surface potential representation showing an electropositive patch formed by RAD51 residues K39, K40, K64, K70 and K73.
(F) Atomic model of the 3′ junction, for comparison with Fig. S10F. In both cases, $RAD51^{F279}$ stacks against the 3′ duplex arm.

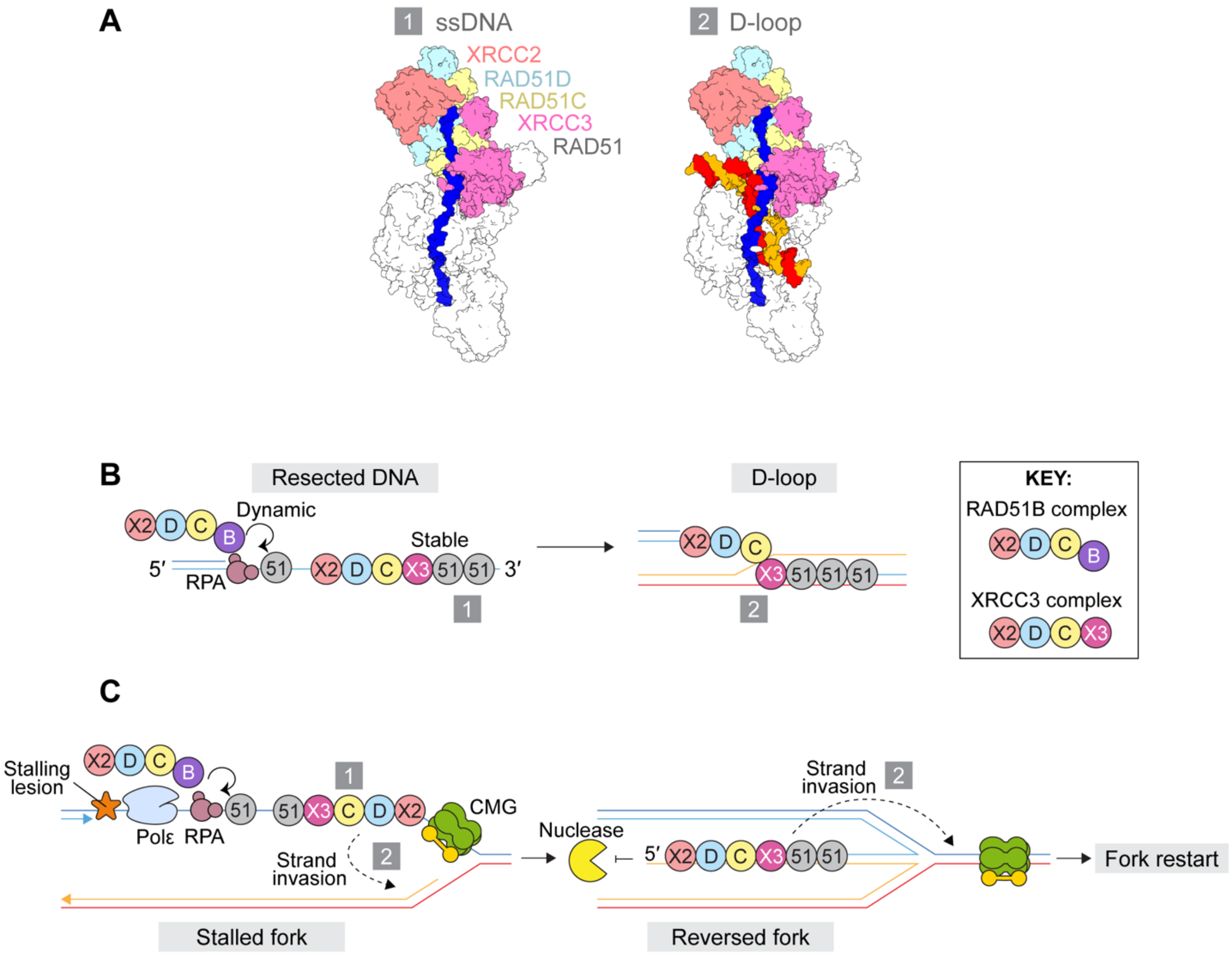
A
1 ssDNA
2 D-loop
XRCC2
RAD51D
RAD51C
XRCC3
RAD51
B
Resected DNA
D-loop
Dynamic
Stable
5′
3′
RPA
X2
D
C
B
X3
51
1
2
KEY:
RAD51B complex
XRCC3 complex
C
Stalling lesion
Polε
RPA
CMG
Strand invasion
Nuclease
5′
Fork restart
Stalled fork
Reversed fork

Fig. S13. Proposed functions of the RAD51 paralogs in homologous recombination and replication stress response

(A) Illustrations show the XRCC3 complex capping RAD51 (1) ssDNA and (2) D-loop filaments.

(B) Proposed role for the RAD51 paralogs in homologous recombination. Following DNA end resection, RPA binds to ssDNA, which acts as a substrate for the assembly of RAD51 filaments by the RAD51B complex. Filaments are (1) capped by the XRCC3 complex, which (2) further assists in strand invasion.

(C) Proposed roles for the RAD51 paralogs in replication stress. Polymerase-helicase uncoupling leads to the accumulation of RPA-bound ssDNA behind the fork, which acts as a substrate for the assembly of RAD51 filaments by the RAD51B complex. Filaments are (1) capped by the XRCC3 complex. The XRCC3 complex may promote (2) RAD51-mediated strand invasion in both fork reversal and restart. Fork remodeling by RAD51, helicases and translocases leads to fork reversal into a four-way ‘chicken-foot’ structure, which may be protected by the XRCC3 complex and RAD51.

Table 1. Cryo-EM data collection, refinement, and validation statistics

	ssDNA<br>9SVX<br>EMDB-55290	Tailed duplex<br>9SVY<br>EMDB-55290	D-loop<br>9SW0<br>EMDB-55293
**Data collection and Processing (for each dataset)**			
Microscope	FEI Titan Krios	FEI Titan Krios	FEI Titan Krios
Voltage (keV)	300	300	300
Camera	Falcon 4i	Falcon 4i	Falcon 4i
Magnification	130,000	130,000	130,000
Pixel size at detector (Å/pixel)	0.95	0.95	0.95
Total electron exposure ($e^-/Å^2$)	40.8	46.3	48.4
Exposure rate (e-/pixel/sec)	6.78	7.69	8.04
Number of frames collected during exposure	1,674 (EER)	1,674 (EER)	1,674 (EER)
Defocus range (μm)	-1.0 to -2.7	-0.8 to -2.3	-0.8 to -2.3
Phase plate (if used)	n/a	n/a	n/a
- phase shift range (in degrees)			
- number of images per phase plate position			
Automation software (EPU, SerialEM or manual)	EPU	EPU	EPU
Tilt angle (if grid was tilted)	n/a	n/a	n/a
Energy filter slit width (if used)	10 eV	10 eV	10 eV
Micrographs collected (no.)	39,812	30,713	45,247
Micrographs used (no.)	39,812	30,713	45,247
Total extracted particles (no.)	12,461,490	24,519,489	35,223,698
**For each reconstruction:**			
Final particles (no.)	323,691	230,120	29,848
Point-group or helical symmetry parameters	-	-	-
Estimated error of translations/rotations (if available)	-	-	-
Resolution (global, Å)			
FSC 0.143 (unmasked/masked)	2.7	2.6	3.0
Resolution range (Å) [min,P25,median,P75,max]	2.1, 2.6, 2.9, 3.5, 7.6	2.1, 2.6, 3.1, 4.6, 31.6	2.6, 3.6, 6, 7.9, 50.2
Map sharpening *B* factor ($Å^2$) / (B factor Range)	-	-	-
Map sharpening methods	EMReady	EMReady	EMReady
**Model composition (for each model)**			
Protein	2,761	3,432	3,415
Ligands	21 (7 ATP, 1 ADP, 8 MG, 5 CA)	27 (9 ATP, 1 ADP, 10 MG, 7 CA)	27 (8 ATP, 1 ADP, 8 MG, 7 CA)
DNA (nucleotides)	21	50	98
**Model Refinement (for each model)**			
Refinement package	Phenix	Phenix	Phenix
- real or reciprocal space	Real	Real	Real
Model-Map scores			
-CCvolume/mask	0.85	0.82	0.83
B factors ($Å^2$)			
Protein residues	115.39	115.76	114.99
Ligands	94.78	90.08	88.1
DNA	78.27	82.94	135.51
R.m.s. deviations from ideal values			
Bond lengths (Å)	0.002	0.003	0.002
Bond angles (°)	0.430	0.459	0.471
**Validation (for each model)**			
MolProbity score	1.15	1.30	1.45
CaBLAM outliers	2.08	2.52	2.36
Clashscore	3.58	4.52	6.34
Poor rotamers (%)	0	0	0
C-beta deviations	N/A	N/A	N/A
EMRinger score (if better than 4 Å resolution)	3.43	3.06	2.91
Ramachandran plot			
Favored (%)	98.28	97.68	97.51
Outliers (%)	0	0	0

References and Notes

1. S. Tye, G. E. Ronson, J. R. Morris, A fork in the road: Where homologous recombination and stalled replication fork protection part ways. *Semin. Cell Dev. Biol.* **113**, 14–26 (2021). doi:10.1016/j.semcdb.2020.07.004 Medline

2. F. E. Benson, A. Stasiak, S. C. West, Purification and characterization of the human Rad51 protein, an analogue of E. coli RecA. *EMBO J.* **13**, 5764–5771 (1994). doi:10.1002/j.1460-2075.1994.tb06914.x Medline

3. P. Baumann, F. E. Benson, S. C. West, Human Rad51 protein promotes ATP-dependent homologous pairing and strand transfer reactions in vitro. *Cell* **87**, 757–766 (1996). doi:10.1016/S0092-8674(00)81394-X Medline

4. P. Sung, Catalysis of ATP-dependent homologous DNA pairing and strand exchange by yeast RAD51 protein. *Science* **265**, 1241–1243 (1994). doi:10.1126/science.8066464 Medline

5. J. Xu, L. Zhao, Y. Xu, W. Zhao, P. Sung, H.-W. Wang, Cryo-EM structures of human RAD51 recombinase filaments during catalysis of DNA-strand exchange. *Nat. Struct. Mol. Biol.* **24**, 40–46 (2017). doi:10.1038/nsmb.3336 Medline

6. W. Liu, Y. Saito, J. Jackson, R. Bhowmick, M. T. Kanemaki, A. Vindigni, D. Cortez, RAD51 bypasses the CMG helicase to promote replication fork reversal. *Science* **380**, 382–387 (2023). doi:10.1126/science.add7328 Medline

7. M. Ito, Y. Fujita, A. Shinohara, Positive and negative regulators of RAD51/DMC1 in homologous recombination and DNA replication. *DNA Repair* **134**, 103613 (2024). doi:10.1016/j.dnarep.2023.103613 Medline

8. A. K. C. Wong, R. Pero, P. A. Ormonde, S. V. Tavtigian, P. L. Bartel, RAD51 interacts with the evolutionarily conserved BRC motifs in the human breast cancer susceptibility gene brca2. *J. Biol. Chem.* **272**, 31941–31944 (1997). doi:10.1074/jbc.272.51.31941 Medline

9. P. L. Chen, C.-F. Chen, Y. Chen, J. Xiao, Z. D. Sharp, W.-H. Lee, The BRC repeats in BRCA2 are critical for RAD51 binding and resistance to methyl methanesulfonate treatment. *Proc. Natl. Acad. Sci. U.S.A.* **95**, 5287–5292 (1998). doi:10.1073/pnas.95.9.5287 Medline

10. T. Thorslund, F. Esashi, S. C. West, Interactions between human BRCA2 protein and the meiosis-specific recombinase DMC1. *EMBO J.* **26**, 2915–2922 (2007). doi:10.1038/sj.emboj.7601739 Medline

11. A. A. Davies, J.-Y. Masson, M. J. McIlwraith, A. Z. Stasiak, A. Stasiak, A. R. Venkitaraman, S. C. West, Role of BRCA2 in control of the RAD51 recombination and DNA repair protein. *Mol. Cell* **7**, 273–282 (2001). doi:10.1016/S1097-2765(01)00175-7 Medline

12. H. Yang, P. D. Jeffrey, J. Miller, E. Kinnucan, Y. Sun, N. H. Thomä, N. Zheng, P.-L. Chen, W.-H. Lee, N. P. Pavletich, BRCA2 function in DNA binding and recombination from a BRCA2-DSS1-ssDNA structure. *Science* **297**, 1837–1848 (2002). doi:10.1126/science.297.5588.1837 Medline

13. A. Carreira, J. Hilario, I. Amitani, R. J. Baskin, M. K. K. Shivji, A. R. Venkitaraman, S. C. Kowalczykowski, The BRC repeats of BRCA2 modulate the DNA-binding selectivity of RAD51. *Cell* **136**, 1032–1043 (2009). doi:10.1016/j.cell.2009.02.019 Medline

14. F. Esashi, V. E. Galkin, X. Yu, E. H. Egelman, S. C. West, Stabilization of RAD51 nucleoprotein filaments by the C-terminal region of BRCA2. *Nat. Struct. Mol. Biol.* **14**, 468–474 (2007). doi:10.1038/nsmb1245 Medline

15. R. B. Jensen, A. Carreira, S. C. Kowalczykowski, Purified human BRCA2 stimulates RAD51-mediated recombination. *Nature* **467**, 678–683 (2010). doi:10.1038/nature09399 Medline

16. M. A. Longo, S. M. Ahmed, Y. Chen, C.-L. Tsai, S. Namjoshi, R. Shen, Z. Ahmed, X. Wang, R. L. Perera, A. Arvai, M. Lee, L. R. Kong, W. Engl, W. S. Ng, Z. W. Zhao, A. R. Venkitaraman, J. A. Tainer, K. Schlacher, BRCA2 C-terminal clamp restructures RAD51 dimers to bind B-DNA for replication fork stability. *Mol. Cell* **85**, 2080–2096.e6 (2025). doi:10.1016/j.molcel.2025.05.010 Medline

17. R. Appleby, L. Joudeh, K. Cobbett, L. Pellegrini, Structural basis for stabilisation of the RAD51 nucleoprotein filament by BRCA2. *Nat. Commun.* **14**, 7003 (2023). doi:10.1038/s41467-023-42830-1 Medline

18. B. Xia, Q. Sheng, K. Nakanishi, A. Ohashi, J. Wu, N. Christ, X. Liu, M. Jasin, F. J. Couch, D. M. Livingston, Control of BRCA2 cellular and clinical functions by a nuclear partner, PALB2. *Mol. Cell* **22**, 719–729 (2006). doi:10.1016/j.molcel.2006.05.022 Medline

19. F. Zhang, Q. Fan, K. Ren, P. R. Andreassen, PALB2 functionally connects the breast cancer susceptibility proteins BRCA1 and BRCA2. *Mol. Cancer Res.* **7**, 1110–1118 (2009). doi:10.1158/1541-7786.MCR-09-0123 Medline

20. A. Carver, T.-Y. Yu, L. A. Yates, T. White, R. Wang, K. Lister, M. Jasin, X. Zhang, Molecular basis of FIGNL1 in dissociating RAD51 from DNA and chromatin. *Science* **387**, 426–431 (2025). doi:10.1126/science.adr7920 Medline

21. A. Meindl, H. Hellebrand, C. Wiek, V. Erven, B. Wappenschmidt, D. Niederacher, M. Freund, P. Lichtner, L. Hartmann, H. Schaal, J. Ramser, E. Honisch, C. Kubisch, H. E. Wichmann, K. Kast, H. Deissler, C. Engel, B. Müller-Myhsok, K. Neveling, M. Kiechle, C. G. Mathew, D. Schindler, R. K. Schmutzler, H. Hanenberg, Germline mutations in breast and ovarian cancer pedigrees establish *RAD51C* as a human cancer susceptibility gene. *Nat. Genet.* **42**, 410–414 (2010). doi:10.1038/ng.569 Medline

22. C. Loveday, C. Turnbull, E. Ramsay, D. Hughes, E. Ruark, J. R. Frankum, G. Bowden, B. Kalmyrzaev, M. Warren-Perry, K. Snape, J. W. Adlard, J. Barwell, J. Berg, A. F. Brady, C. Brewer, G. Brice, C. Chapman, J. Cook, R. Davidson, A. Donaldson, F. Douglas, L. Greenhalgh, A. Henderson, L. Izatt, A. Kumar, F. Lalloo, Z. Miedzybrodzka, P. J. Morrison, J. Paterson, M. Porteous, M. T. Rogers, S. Shanley, L. Walker, D. Eccles, D. G. Evans, A. Renwick, S. Seal, C. J. Lord, A. Ashworth, J. S. Reis-Filho, A. C. Antoniou, N. Rahman; Breast Cancer Susceptibility Collaboration (UK), Germline mutations in *RAD51D* confer susceptibility to ovarian cancer. *Nat. Genet.* **43**, 879–882 (2011). doi:10.1038/ng.893 Medline

23. L. Golmard, V. Caux-Moncoutier, G. Davy, E. Al Ageeli, B. Poirot, C. Tirapo, D. Michaux, C. Barbaroux, C. D. d'Enghien, A. Nicolas, L. Castéra, X. Sastre-Garau, M.-H. Stern, C. Houdayer, D. Stoppa-Lyonnet, Germline mutation in the *RAD51B* gene confers predisposition to breast cancer. *BMC Cancer* **13**, 484 (2013). doi:10.1186/1471-2407-13-484 Medline

24. L. Golmard, L. Castéra, S. Krieger, V. Moncoutier, K. Abidallah, H. Tenreiro, A. Laugé, J. Tarabeux, G. A. Millot, A. Nicolas, M. Laé, C. Abadie, P. Berthet, F. Polycarpe, T. Frébourg, C. Elan, A. de Pauw, M. Gauthier-Villars, B. Buecher, M.-H. Stern, D. Stoppa-Lyonnet, D. Vaur, C. Houdayer, Contribution of germline deleterious variants in the RAD51 paralogs to breast and ovarian cancers. *Eur. J. Hum. Genet.* **25**, 1345–1353 (2017). doi:10.1038/s41431-017-0021-2 Medline

25. J. Y. Park, E. L. Virts, A. Jankowska, C. Wiek, M. Othman, S. C. Chakraborty, G. H. Vance, F. S. Alkuraya, H. Hanenberg, P. R. Andreassen, Complementation of hypersensitivity to DNA interstrand crosslinking agents demonstrates that *XRCC2* is a Fanconi anaemia gene. *J. Med. Genet.* **53**, 672–680 (2016). doi:10.1136/jmedgenet-2016-103847 Medline

26. F. Vaz, H. Hanenberg, B. Schuster, K. Barker, C. Wiek, V. Erven, K. Neveling, D. Endt, I. Kesterton, F. Autore, F. Fraternali, M. Freund, L. Hartmann, D. Grimwade, R. G. Roberts, H. Schaal, S. Mohammed, N. Rahman, D. Schindler, C. G. Mathew, Mutation of the *RAD51C* gene in a Fanconi anemia-like disorder. *Nat. Genet.* **42**, 406–409 (2010). doi:10.1038/ng.570 Medline

27. R. Prakash, L. Freyer, N. Saiz, S. Gavrilov, R. Q. Wang, P. J. Romanienko, E. Lacy, A.-K. Hadjantonakis, M. Jasin, XRCC3 loss leads to midgestational embryonic lethality in mice. *DNA Repair* **108**, 103227 (2021). doi:10.1016/j.dnarep.2021.103227 Medline

28. S. G. Kuznetsov, D. C. Haines, B. K. Martin, S. K. Sharan, Loss of Rad51c leads to embryonic lethality and modulation of Trp53-dependent tumorigenesis in mice. *Cancer Res.* **69**, 863–872 (2009). doi:10.1158/0008-5472.CAN-08-3057 Medline

29. D. L. Pittman, J. C. Schimenti, Midgestation lethality in mice deficient for the *RecA*-related gene, *Rad51d*/*Rad51l3*. *Genesis* **26**, 167–173 (2000). doi:10.1002/(SICI)1526-968X(200003)26:3<167:AID-GENE1>3.0.CO;2-M Medline

30. Z. Shu, S. Smith, L. Wang, M. C. Rice, E. B. Kmiec, Disruption of *muREC2*/*RAD51L1* in mice results in early embryonic lethality which can Be partially rescued in a *p53(-/-)* background. *Mol. Cell. Biol.* **19**, 8686–8693 (1999). doi:10.1128/MCB.19.12.8686 Medline

31. B. Deans, C. S. Griffin, M. Maconochie, J. Thacker, *Xrcc2* is required for genetic stability, embryonic neurogenesis and viability in mice. *EMBO J.* **19**, 6675–6685 (2000). doi:10.1093/emboj/19.24.6675 Medline

32. E. B. Garcin, S. Gon, M. R. Sullivan, G. J. Brunette, A. Cian, J.-P. Concordet, C. Giovannangeli, W. G. Dirks, S. Eberth, K. A. Bernstein, R. Prakash, M. Jasin, M. Modesti, Differential requirements for the RAD51 paralogs in genome repair and maintenance in human cells. *PLOS Genet.* **15**, e1008355 (2019). doi:10.1371/journal.pgen.1008355 Medline

33. M. Berti, F. Teloni, S. Mijic, S. Ursich, J. Fuchs, M. D. Palumbieri, J. Krietsch, J. A. Schmid, E. B. Garcin, S. Gon, M. Modesti, M. Altmeyer, M. Lopes, Sequential role of RAD51 paralog complexes in replication fork remodeling and restart. *Nat. Commun.* **11**, 3531 (2020). doi:10.1038/s41467-020-17324-z Medline

34. E. Petermann, M. L. Orta, N. Issaeva, N. Schultz, T. Helleday, Hydroxyurea-stalled replication forks become progressively inactivated and require two different RAD51-

mediated pathways for restart and repair. *Mol. Cell* **37**, 492–502 (2010). doi:10.1016/j.molcel.2010.01.021 Medline

35. R. Zellweger, D. Dalcher, K. Mutreja, M. Berti, J. A. Schmid, R. Herrador, A. Vindigni, M. Lopes, Rad51-mediated replication fork reversal is a global response to genotoxic treatments in human cells. *J. Cell Biol.* **208**, 563–579 (2015). doi:10.1083/jcb.201406099 Medline
36. M. A. Longo, S. Roy, Y. Chen, K.-H. Tomaszowski, A. S. Arvai, J. T. Pepper, R. A. Boisvert, S. Kunnimalaiyaan, C. Keshvani, D. Schild, A. Bacolla, G. J. Williams, J. A. Tainer, K. Schlacher, RAD51C-XRCC3 structure and cancer patient mutations define DNA replication roles. *Nat. Commun.* **14**, 4445 (2023). doi:10.1038/s41467-023-40096-1 Medline
37. J.-Y. Masson, M. C. Tarsounas, A. Z. Stasiak, A. Stasiak, R. Shah, M. J. McIlwraith, F. E. Benson, S. C. West, Identification and purification of two distinct complexes containing the five RAD51 paralogs. *Genes Dev.* **15**, 3296–3307 (2001). doi:10.1101/gad.947001 Medline
38. L. A. Greenhough, C.-C. Liang, O. Belan, S. Kunzelmann, S. Maslen, M. C. Rodrigo-Brenni, R. Anand, M. Skehel, S. J. Boulton, S. C. West, Structure and function of the RAD51B-RAD51C-RAD51D-XRCC2 tumour suppressor. *Nature* **619**, 650–657 (2023). doi:10.1038/s41586-023-06179-1 Medline
39. Y. Rawal, L. Jia, A. Meir, S. Zhou, H. Kaur, E. A. Ruben, Y. Kwon, K. A. Bernstein, M. Jasin, A. B. Taylor, S. Burma, R. Hromas, A. V. Mazin, W. Zhao, D. Zhou, E. V. Wasmuth, E. C. Greene, P. Sung, S. K. Olsen, Structural insights into BCDX2 complex function in homologous recombination. *Nature* **619**, 640–649 (2023). doi:10.1038/s41586-023-06219-w Medline
40. M. Akita, P. Girvan, M. Spirek, J. Novacek, D. Rueda, Z. Prokop, L. Krejci, Mechanism of BCDX2-mediated RAD51 nucleation on short ssDNA stretches and fork DNA. *Nucleic Acids Res.* **52**, 11738–11752 (2024). doi:10.1093/nar/gkae770 Medline
41. J.-Y. Masson, A. Z. Stasiak, A. Stasiak, F. E. Benson, S. C. West, Complex formation by the human RAD51C and XRCC3 recombination repair proteins. *Proc. Natl. Acad. Sci. U.S.A.* **98**, 8440–8446 (2001). doi:10.1073/pnas.111005698 Medline
42. J. Abramson, J. Adler, J. Dunger, R. Evans, T. Green, A. Pritzel, O. Ronneberger, L. Willmore, A. J. Ballard, J. Bambrick, S. W. Bodenstein, D. A. Evans, C.-C. Hung, M. O'Neill, D. Reiman, K. Tunyasuvunakool, Z. Wu, A. Žemgulytė, E. Arvaniti, C. Beattie, O. Bertolli, A. Bridgland, A. Cherepanov, M. Congreve, A. I. Cowen-Rivers, A. Cowie, M. Figurnov, F. B. Fuchs, H. Gladman, R. Jain, Y. A. Khan, C. M. R. Low, K. Perlin, A. Potapenko, P. Savy, S. Singh, A. Stecula, A. Thillaisundaram, C. Tong, S. Yakneen, E. D. Zhong, M. Zielinski, A. Žídek, V. Bapst, P. Kohli, M. Jaderberg, D. Hassabis, J. M. Jumper, Accurate structure prediction of biomolecular interactions with AlphaFold 3. *Nature* **630**, 493–500 (2024). doi:10.1038/s41586-024-07487-w Medline
43. Z. Lin, H. Kong, M. Nei, H. Ma, Origins and evolution of the *recA*/*RAD51* gene family: Evidence for ancient gene duplication and endosymbiotic gene transfer. *Proc. Natl. Acad. Sci. U.S.A.* **103**, 10328–10333 (2006). doi:10.1073/pnas.0604232103 Medline

44. H. Yang, C. Zhou, A. Dhar, N. P. Pavletich, Mechanism of strand exchange from RecA-DNA synaptic and D-loop structures. *Nature* **586**, 801–806 (2020). doi:10.1038/s41586-020-2820-9 Medline

45. L. Joudeh, R. E. Appleby, J. D. Maman, L. Pellegrini, Structural mechanism of strand exchange by the RAD51 filament. *eLife* **14**, RP107114 (2025). doi:10.7554/eLife.107114.3 Medline

46. M.-C. Ho, Z.-C. Luo, C.-H. Yang, H.-Y. Yeh, C.-W. Lin, M.-C. Yeh, P. Chi, RAD51 D-loop structures reveal the mechanism of eukaryotic RAD51-mediated strand exchange. *Nat. Portfolio* (2025); https://doi.org/10.21203/rs.3.rs-6511672/v1.

47. C. Richardson, N. Horikoshi, T. K. Pandita, The role of the DNA double-strand break response network in meiosis. *DNA Repair* **3**, 1149–1164 (2004). doi:10.1016/j.dnarep.2004.05.007 Medline

48. J. M. Mason, K. Dusad, W. D. Wright, J. Grubb, B. Budke, W.-D. Heyer, P. P. Connell, R. R. Weichselbaum, D. K. Bishop, RAD54 family translocases counter genotoxic effects of RAD51 in human tumor cells. *Nucleic Acids Res.* **43**, 3180–3196 (2015). doi:10.1093/nar/gkv175 Medline

49. P. Sung, Yeast Rad55 and Rad57 proteins form a heterodimer that functions with replication protein A to promote DNA strand exchange by Rad51 recombinase. *Genes Dev.* **11**, 1111–1121 (1997). doi:10.1101/gad.11.9.1111 Medline

50. K. Somyajit, S. Saxena, S. Babu, A. Mishra, G. Nagaraju, Mammalian RAD51 paralogs protect nascent DNA at stalled forks and mediate replication restart. *Nucleic Acids Res.* **43**, 9835–9855 (2015). doi:10.1093/nar/gkv880 Medline

51. T. S. Byun, M. Pacek, M. C. Yee, J. C. Walter, K. A. Cimprich, Functional uncoupling of MCM helicase and DNA polymerase activities activates the ATR-dependent checkpoint. *Genes Dev.* **19**, 1040–1052 (2005). doi:10.1101/gad.1301205 Medline

52. Y. Hashimoto, A. Ray Chaudhuri, M. Lopes, V. Costanzo, Rad51 protects nascent DNA from Mre11-dependent degradation and promotes continuous DNA synthesis. *Nat. Struct. Mol. Biol.* **17**, 1305–1311 (2010). doi:10.1038/nsmb.1927 Medline

53. K. Schlacher, N. Christ, N. Siaud, A. Egashira, H. Wu, M. Jasin, Double-strand break repair-independent role for BRCA2 in blocking stalled replication fork degradation by MRE11. *Cell* **145**, 529–542 (2011). doi:10.1016/j.cell.2011.03.041 Medline

54. C. Elfmann, J. Stülke, PAE viewer: A webserver for the interactive visualization of the predicted aligned error for multimer structure predictions and crosslinks. *Nucleic Acids Res.* **51** (W1), W404–W410 (2023). doi:10.1093/nar/gkad350 Medline

55. D. M. Emms, S. Kelly, OrthoFinder: Phylogenetic orthology inference for comparative genomics. *Genome Biol.* **20**, 238 (2019). doi:10.1186/s13059-019-1832-y Medline

56. K. Katoh, D. M. Standley, MAFFT multiple sequence alignment software version 7: Improvements in performance and usability. *Mol. Biol. Evol.* **30**, 772–780 (2013). doi:10.1093/molbev/mst010 Medline

57. B. Q. Minh, H. A. Schmidt, O. Chernomor, D. Schrempf, M. D. Woodhams, A. von Haeseler, R. Lanfear, IQ-TREE 2: New models and efficient methods for phylogenetic

inference in the genomic era. *Mol. Biol. Evol.* **37**, 1530–1534 (2020). doi:10.1093/molbev/msaa015 Medline

58. K. Sano, K. Maeda, M. Oki, Y. Maéda, Enhancement of protein expression in insect cells by a lobster tropomyosin cDNA leader sequence. *FEBS Lett.* **532**, 143–146 (2002). doi:10.1016/S0014-5793(02)03659-1 Medline

59. A. Benitez, M. Sebald, R. Kanagaraj, M. C. Rodrigo-Brenni, Y. W. Chan, C.-C. Liang, S. C. West, GEN1 promotes common fragile site expression. *Cell Rep.* **42**, 112062 (2023). doi:10.1016/j.celrep.2023.112062 Medline

60. F. Weissmann, G. Petzold, R. VanderLinden, P. J. Huis In 't Veld, N. G. Brown, F. Lampert, S. Westermann, H. Stark, B. A. Schulman, J.-M. Peters, biGBac enables rapid gene assembly for the expression of large multisubunit protein complexes. *Proc. Natl. Acad. Sci. U.S.A.* **113**, E2564–E2569 (2016). doi:10.1073/pnas.1604935113 Medline

61. C.-C. Liang, L. A. Greenhough, L. Masino, S. Maslen, I. Bajrami, M. Tuppi, M. Skehel, I. A. Taylor, S. C. West, Mechanism of single-stranded DNA annealing by RAD52-RPA complex. *Nature* **629**, 697–703 (2024). doi:10.1038/s41586-024-07347-7 Medline

62. O. Belan, C. Barroso, A. Kaczmarczyk, R. Anand, S. Federico, N. O'Reilly, M. D. Newton, E. Maeots, R. I. Enchev, E. Martinez-Perez, D. S. Rueda, S. J. Boulton, Single-molecule analysis reveals cooperative stimulation of Rad51 filament nucleation and growth by mediator proteins. *Mol. Cell* **81**, 1058–1073.e7 (2021). doi:10.1016/j.molcel.2020.12.020 Medline

63. A. Candelli, J. T. Holthausen, M. Depken, I. Brouwer, M. A. M. Franker, M. Marchetti, I. Heller, S. Bernard, E. B. Garcin, M. Modesti, C. Wyman, G. J. L. Wuite, E. J. G. Peterman, Visualization and quantification of nascent RAD51 filament formation at single-monomer resolution. *Proc. Natl. Acad. Sci. U.S.A.* **111**, 15090–15095 (2014). doi:10.1073/pnas.1307824111 Medline

64. D. Kimanius, L. Dong, G. Sharov, T. Nakane, S. H. W. Scheres, New tools for automated cryo-EM single-particle analysis in RELION-4.0. *Biochem. J.* **478**, 4169–4185 (2021). doi:10.1042/BCJ20210708 Medline

65. A. Punjani, J. L. Rubinstein, D. J. Fleet, M. A. Brubaker, cryoSPARC: Algorithms for rapid unsupervised cryo-EM structure determination. *Nat. Methods* **14**, 290–296 (2017). doi:10.1038/nmeth.4169 Medline

66. S. Q. Zheng, E. Palovcak, J.-P. Armache, K. A. Verba, Y. Cheng, D. A. Agard, MotionCor2: Anisotropic correction of beam-induced motion for improved cryo-electron microscopy. *Nat. Methods* **14**, 331–332 (2017). doi:10.1038/nmeth.4193 Medline

67. A. Rohou, N. Grigorieff, CTFFIND4: Fast and accurate defocus estimation from electron micrographs. *J. Struct. Biol.* **192**, 216–221 (2015). doi:10.1016/j.jsb.2015.08.008 Medline

68. T. Bepler, A. Morin, M. Rapp, J. Brasch, L. Shapiro, A. J. Noble, B. Berger, Positive-unlabeled convolutional neural networks for particle picking in cryo-electron micrographs. *Nat. Methods* **16**, 1153–1160 (2019). doi:10.1038/s41592-019-0575-8 Medline

69. J. He, T. Li, S. Y. Huang, Improvement of cryo-EM maps by simultaneous local and non-local deep learning. *Nat. Commun.* **14**, 3217 (2023). doi:10.1038/s41467-023-39031-1 Medline

70. A. Punjani, D. J. Fleet, 3DFlex: Determining structure and motion of flexible proteins from cryo-EM. *Nat. Methods* **20**, 860–870 (2023). doi:10.1038/s41592-023-01853-8 Medline

71. A. Punjani, H. Zhang, D. J. Fleet, Non-uniform refinement: Adaptive regularization improves single-particle cryo-EM reconstruction. *Nat. Methods* **17**, 1214–1221 (2020). doi:10.1038/s41592-020-00990-8 Medline

72. E. F. Pettersen, T. D. Goddard, C. C. Huang, E. C. Meng, G. S. Couch, T. I. Croll, J. H. Morris, T. E. Ferrin, UCSF ChimeraX: Structure visualization for researchers, educators, and developers. *Protein Sci.* **30**, 70–82 (2021). doi:10.1002/pro.3943 Medline

73. R. Appleby, D. Bollschweiler, D. Y. Chirgadze, L. Joudeh, L. Pellegrini, A metal ion-dependent mechanism of RAD51 nucleoprotein filament disassembly. *iScience* **26**, 106689 (2023). doi:10.1016/j.isci.2023.106689 Medline

74. A. Casañal, B. Lohkamp, P. Emsley, Current developments in Coot for macromolecular model building of Electron Cryo-microscopy and Crystallographic Data. *Protein Sci.* **29**, 1069–1078 (2020). doi:10.1002/pro.3791 Medline

75. T. I. Croll, ISOLDE: A physically realistic environment for model building into low-resolution electron-density maps. *Acta Crystallogr. D Struct. Biol.* **74**, 519–530 (2018). doi:10.1107/S2059798318002425 Medline

76. D. Liebschner, P. V. Afonine, M. L. Baker, G. Bunkóczi, V. B. Chen, T. I. Croll, B. Hintze, L.-W. Hung, S. Jain, A. J. McCoy, N. W. Moriarty, R. D. Oeffner, B. K. Poon, M. G. Prisant, R. J. Read, J. S. Richardson, D. C. Richardson, M. D. Sammito, O. V. Sobolev, D. H. Stockwell, T. C. Terwilliger, A. G. Urzhumtsev, L. L. Videau, C. J. Williams, P. D. Adams, Macromolecular structure determination using X-rays, neutrons and electrons: Recent developments in Phenix. *Acta Crystallogr. D Struct. Biol.* **75**, 861–877 (2019). doi:10.1107/S2059798319011471 Medline
